# Speciation of pelagic zooplankton: Invisible boundaries can drive isolation of oceanic ctenophores

**DOI:** 10.3389/fgene.2022.970314

**Published:** 2022-10-07

**Authors:** Shannon B. Johnson, Jacob R. Winnikoff, Darrin T. Schultz, Lynne M. Christianson, Wyatt L. Patry, Claudia E. Mills, Steven H. D. Haddock

**Affiliations:** ^1^ Monterey Bay Aquarium Research Institute, Moss Landing, CA, United States; ^2^ Department of Ecology and Evolutionary Biology, University of California, Santa Cruz, Santa Cruz, CA, United States; ^3^ Department of Neurosciences and Developmental Biology, University of Vienna, Vienna, Austria; ^4^ Department of Biomolecular Engineering and Bioinformatics, University of California, Santa Cruz, Santa Cruz, CA, United States; ^5^ Animal Care Division, Monterey Bay Aquarium, Monterey, CA, United States; ^6^ Friday Harbor Laboratories and the Department of Biology, University of Washington, Friday Harbor, WA, United States

**Keywords:** ctenophora, comb jelly, Bolinopsis, population genomics, cytonuclear discordance, new species, zooplankton

## Abstract

The study of evolution and speciation in non-model systems provides us with an opportunity to expand our understanding of biodiversity in nature. Connectivity studies generally focus on species with obvious boundaries to gene flow, but in open-ocean environments, such boundaries are difficult to identify. Due to the lack of obvious boundaries, speciation and population subdivision in the pelagic environment remain largely unexplained. Comb jellies (Phylum Ctenophora) are mostly planktonic gelatinous invertebrates, many of which are considered to have freely interbreeding distributions worldwide. It is thought that the lobate ctenophore *Bolinopsis infundibulum* is distributed throughout cooler northern latitudes and *B. vitrea* warmer. Here, we examined the global population structure for species of *Bolinopsis* with genetic and morphological data. We found distinct evolutionary patterns within the genus, where *B. infundibulum* had a broad distribution from northern Pacific to Atlantic waters despite many physical barriers, while other species were geographically segregated despite few barriers. Divergent patterns of speciation within the genus suggest that oceanic currents, sea-level, and geological changes over time can act as either barriers or aids to dispersal in the pelagic environment. Further, we used population genomic data to examine evolution in the open ocean of a distinct lineage of *Bolinopsis* ctenophores from the North Eastern Pacific. Genetic information and morphological observations validated this as a separate species, *Bolinopsis microptera*, which was previously described but has recently been called *B. infundibulum*. We found that populations of *B. microptera* from California were in cytonuclear discordance, which indicates a secondary contact zone for previously isolated populations. Discordance at this scale is rare, especially in a continuous setting.

## Introduction

The midwater zone of the world ocean is an enormous region of seemingly limitless connectivity inhabited by mostly holoplanktonic species, which spend their entire lives drifting or swimming in the water column (Robison, 2009). Species with such “open” populations are generally thought to have less genetic structure, because they have high effective population sizes and therefore are less subject to genetic drift (Hellberg et al., 2002; Peijnenburg and Goetze, 2013). Due to perceived lack of barriers, lower species diversity than the benthos, large effective population sizes, and high rates of dispersal, it is thought that many pelagic species have global population distributions (Peijnenburg and Goetze, 2013). However, results from the few prior studies of zooplankton population genetics reveal large amounts of population structure among ocean basins and even, surprisingly, on smaller scales (Peijnenburg and Goetze, 2013; Verwimp et al., 2019; Jaspers et al., 2021). It has been proposed that selection, rather than genetic drift, is the dominant driver in evolution of oceanic zooplankton species (Peijnenburg and Goetze, 2013).

Ctenophora is a phylum of gelatinous marine predators characterized by eight longitudinal rows of ciliary comb plates along their bodies, which function as paddles for locomotion (Tamm, 2014). There are ∼200 described and many yet-undescribed species of ctenophores (Haddock, 2004) that occupy all areas of the world ocean (Mills, 2020). A trait common to nearly all species in the phylum is simultaneous hermaphroditism with the capacity to self-fertilize (Dunn et al., 2015). Apart from the Platyctenida, an order of benthic ctenophores, and potentially the genus *Lobatolampea,* comb jellies are holoplanktonic. Although most species of ctenophores are thought to be weak swimmers, they are expected to disperse long distances during their lifespan, propelled mostly by ocean currents (Bayha et al., 2014).

The family Bolinopsidae (Ctenophora: Lobata) includes the well-studied coastal species, *Mnemiopsis leidyi,* which is paraphyletic within the *Bolinopsis* genus (Christianson et al., 2022). *Bolinopsis* are oceanic ctenophores that are common and seasonally abundant from the surface to nearly 2,000 m depth and are highly fecund simultaneous hermaphrodites (Dunlap-Pianka, 1974; Strathmann, 1987). There are currently nine named species within the genus (Mills, 2020; https://www.marinespecies.org/aphia.php?p=taxdetails&id=106350), some of which have been synonymized. *Bolinopsis infundibulum* (O.F. Müller, 1776) is a large species thought to have a global distribution that encompasses most of the North Pacific, Arctic, and N. Atlantic oceans. *Bolinopsis vitrea* (L. Agassiz, 1860) is thought to occupy tropical and subtropical regions of the Eastern Atlantic Ocean and the Black Sea (Mayer, 1912; Harbison et al., 1978). In 1912, Mortensen synonymized the temperate Pacific *Bolinopsis microptera* with *B. infundibulum,* although the former name has remained in occasional use (Light, 1954; Dunlap, 1966; Dunlap-Pianka, 1974; Freeman, 1977; Lucas and Dawson, 2014; El-Bawab, 2020).

With the exception of *Mnemiopsis leidyi* (Bayha et al., 2014; Jaspers et al., 2021), the global distribution of very few ctenophores have been examined, particularly not for deep-sea oceanic species. We determined the biogeographic structure of species and populations within the genus *Bolinopsis*, first with the barcoding fragment of mitochondrial cytochrome C-oxidase subunit I (*COI*) with newly designed primers (Christianson et al., 2022). Our results highlighted different genetic patterns within the genus. For example, despite many physical barriers, *B. infundibulum* sensu strictu (s.s.) truly had a broad distribution that spanned the Arctic from the Bering Sea to the Northern Atlantic. In contrast, we found differentiation among all the other lineages we sequenced, even where no barriers were evident. The present work confirms that the name *Bolinopsis microptera* is appropriately applied to *Bolinopsis* from Washington State to California*,* and we formally reverse the proposed synonymy (Mortensen, 1912) of *B. microptera* with *B. infundibulum.* We have illustrated a specimen used for sequencing to provide a morphological re-description for *Bolinopsis microptera* (SI Supplementary Appendix A1) and use the name *B. microptera* for the remainder of the manuscript.

To explore patterns of speciation and evolution of pelagic ctenophores we also studied population genomics of *B. microptera.* Here we found cytonuclear discordance, where California represented a massive secondary contact zone between previously isolated populations. Our results suggest a sharp contrast to the idea that pelagic organisms have cosmopolitan distributions and few boundaries to gene flow. Midwater communities may change their composition and distributions as a result of environmental change and the resulting changes in circulation patterns, an expanding oxygen minimum zone, and direct human activities such as pollution and deep-sea mining (Drazen et al., 2020). To detect these changes, it is critical that we document the hidden biodiversity of the world ocean’s midwater region, which is the largest habitat on the planet, and remains underexplored both visually and genetically.

## Materials and methods

### Ctenophore sample acquisition

Samples from shallower than 20 m were collected by hand while blue-water SCUBA diving, as well as from the Friday Harbor Laboratories dock. Deeper samples were obtained using remotely operated vehicles *Tiburon, Ventana* or *Doc Ricketts* (Table 1). Because ctenophores do not preserve well in ethanol or formaldehyde, specimens were carefully photographed upon collection. Ctene rows and the underlying canals were then removed with clean plastic transfer pipettes, aliquoted into 2 ml cryotubes, flash frozen in liquid nitrogen, and transferred to a −80°C freezer for storage. Special care was taken to avoid the gut and its contents during dissection.

**TABLE 1:** Samples of *Bolinopsis* species used in morphological and genetic analyses, their provenance, and depths. Sample size of individuals sequenced for *COI* fragment indicated by N.

Locality	Species	Lat	Lon	Depth (m)	N
Alaska	*B. infundibulum* s.s.	56.349	−161.587	10	2
Australia	*B. ashleyi*,	−23.449	151.913,	10	2
	*Bolinopsis* n. sp.	−23.477	151.962		
Bahamas	*B. vitrea*	26.10	−77.43	10	3
Eastern Greenland	*B. infundibulum* s.s.	70.480	−21.943	10	1
Friday Harbor, WA	*B. microptera*	48.546	−123.013	10	34
Kona, Hawaii	*B.* aff. *vitrea*	19.618	−156.124	10	17
Miami, Florida	*Mnemiopsis leidyi*	25.788	−80.059	10	2
Moorea, Tahiti	*B.* aff. *vitrea*	−17.539	−149.830	10	1
Monterey Bay, CA	*B. microptera*	36.600	−122.547300	10–1768	138
MBA	WAxCA				192
N. Atlantic	*B. infundibulum* s.s.	41.0	−689.0	10	3
Santa Cruz Basin, CA	*B. microptera*	33.85	−119.98	1,615	1
Santa Lucia Knoll, CA	*B. microptera*	34.56	−122.55	10-1850	16
San Luis Obispo, CA	*B. microptera*	35.216	−121.332	436	2
Svalbard, Norway	*B. infundibulum* s.s.	79.691	14.010	10	3
Tokyo Bay, Japan	*B. mikado*	35.531	139.889	10	5
Western Iceland	*B. infundibulum* s.s.	65.494	−24.402	10	5

### Sanger sequencing and statistical methods for Bolinopsidae

Methods for our initial targeted Sanger sequencing investigation are detailed in SI Supplementary Appendix A2 and Supplementary Table S1.

### Morphometric measurements and seasonal abundance of Bolinopsidae

**FIGURE 1:**
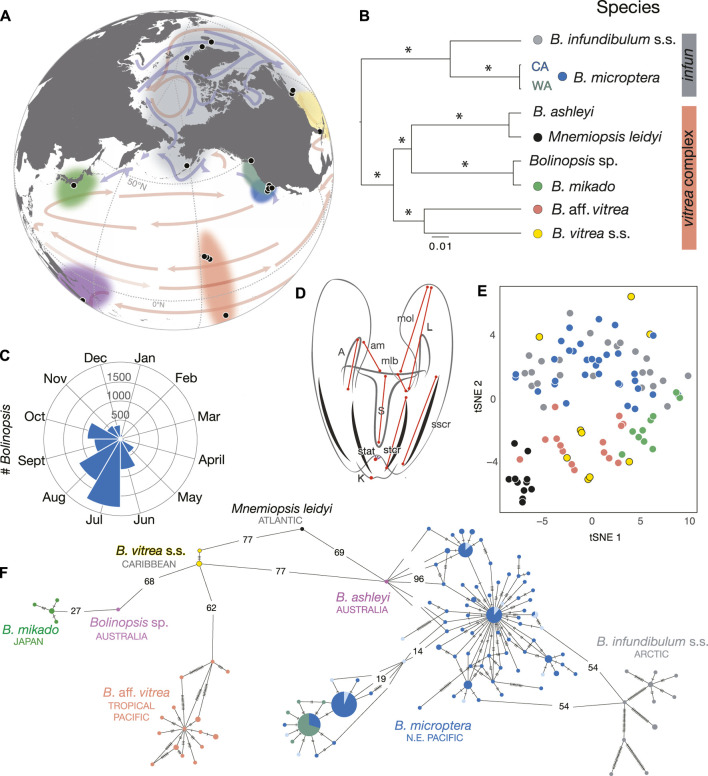
**(A)** Worldwide sampling localities for specimens sequenced from the genera *Bolinopsis* and *Mnemiopsis,* species designated by colors at nodes of species tree **(B)**. **(B)** Species tree estimated with the GTR + I+Γ for *Bolinopsis* species + *M. leidyi* with fragments of COI (648 bp)*, H3* (352 bp)*,* and 28S (996 bp) including *B. infundibulum* (gray bar) and *B. vitrea* (coral bar) species complexes. * indicates 1.0 posterior probability. **(C)** Seasonal distribution of California *Bolinopsis*, visualized by ROV videos at MBARI by midwater research groups. **(D)** Illustration of *Bolinopsis* showing landmarks used in tSNE analysis including auricle (A) and lobe (L) lengths, the distances from the mouth to the oral lobe (mol), mouth to the lobe base (mlb), keel (K), statocyst (stat), the auricle tip to the mouth (am), and the lengths of the sub-tentacular ctene row (stcr) and sub-stomodaeal ctene row (sscr), all of which were normalized by the length of the stomodaeum (S). **(E)** tSNE plot of combined morphological measurements designated in **(D)** of *M. leidyi* and *Bolinopsis* species, colors correspond to species in **(B)** and **(F)**. **(F)** Corresponding minimum spanning network of 259 individuals for a 648 bp fragment of mitochondrial *COI.* Dashes or numbers on branches represent mutations.

In order to provide a useful morphological re-description of *B. microptera*, we measured morphological features that might be used to distinguish between six species of *Bolinopsis* and *Mnemiopsis* (Figure 1D). We included *M. leidyi* since it forms a clade with *B. vitrea* s.s.*, B.* aff. *vitrea,* and *B. mikado* (Figure 1B, Christianson et al., 2022) We digitized landmarks on photographs with the program StereoMorph v.1.6.3 (Olsen and Westneat, 2015) in R v.4.1.0 (R Team, 2017) with Rstudio v.1.4.1717 (Rstudio Team, 2015), including mouth, tentacle bulb, length and width of the stomodaeum, lengths of the infundibulum, statocyst, aboral keel, lobe, and auricle. We acquired 26 images of *B. infundibulum* s.s. and 10 images of *M. leidyi* from iNaturalist.org observations in areas that were unique for the taxa (i.e., Norway, Arctic, and N. Atlantic for *B. infundibulum* s.s.). We included eight more images of *B. infundibulum* s.s. photographed by Erland Svensen in Norway. We included seven of our own images of *B. microptera* each from Friday Harbor, WA and San Luis Obispo, CA, and 19 images of *B. microptera* from the Monterey Bay area, CA. We also included 11 images of *B. mikado* from Tokyo Bay, Japan, 15 images of *B.* aff. *vitrea* from Hawaii, nine images of *B. vitrea* s.s. from Florida (photographed by Richard Collins) and two of our own from the Bahamas. We read the landmarks with the geomorph package v.4.0.0 (Baken et al., 1997; Adams et al., 2021) in R, calculated the inter-landmark distances and normalized measurements by stomodaeum length (mouth to infundibulum). We estimated t-distributed stochastic neighbor embedding (t-SNE) with the package Rtsne v.0.15 (Krijthe, 2015) in R with a perplexity level of 20 with 1,000 iterations to compare the normalized inter-landmark distances and lengths of auricle, lobe, mouth to oral tip of lobe, mouth to lobe base, mouth to aboral keel, statocyst to aboral keel, auricle tip to mouth, sub-tentacular ctene row length, and sub-stomodaeal ctene row among *B. infundibulum* s.s.*, B. microptera*, *B. vitrea* s.s.*, B.* aff. *vitrea* (Hawaii/Moorea), *B. mikado,* and *Mnemiopsis leidyi.* Results were plotted in the Tidyverse (Wickham et al., 2019).

We also plotted seasonal abundance based on unique observations of *B. microptera* from the eastern Pacific from ROV footage from 1989 to 2020 by year (Choy et al., 2017).

### Captive rearing of *B. microptera*

In order to test reproductive incompatibility and potential hybridization between eastern Pacific *Bolinopsis* mitochondrial lineages, we performed a captive spawning experiment. Cultures of *B. microptera* from both Monterey Bay (CA) and Friday Harbor Labs (WA) were maintained at the Monterey Bay Aquarium (MBA). Five individuals of F2 CA and three individuals of wild-caught WA were placed in a wide diffusion tube (Patry et al., 2020; Table 1) for one hour. The spawning time was reduced from 2 h to avoid self-fertilization in the CA specimens as sperm release in the WA population was previously observed to occur much later than that of the CA population. The parents were removed after one hour and sampled. Hatching began 48 h later and larvae were grown on a diet of *Parvocalanus crassirostris* nauplii (Reed Mariculture). Samples of larvae were taken at 17- and 24-days post-hatching for DNA sequencing.

### Tests for hybridization within *B. microptera* using Sanger data

In order to test for reproductive incompatibility and/or hybridization, we individually Sanger-sequenced two batches (collected 1 week apart) of the progeny from the captive spawning experiment (WA×CA) for the following loci: mitochondrial *COI*, nuclear *H3, MDH, LDH1, LDH2,* and *PK* (SI Supplementary Appendix A2; Supplementary Tables S1, S2). To test for admixture and hybridization between the distinct lineages, we used phased allelic data from progeny in STRUCTURE and BA3 analyses comparing WA×CA with wild populations. We used GenoDive v.3.05 to estimate gene flow (F_ST_), inbreeding (G_IS_), individual population assignment (parentage), and a hybrid index for wild eastern Pacific populations and experimental progeny based on allelic data. The hybrid index is an estimated likelihood that the progeny had a higher affinity to one parent population. Since it is haploid, the *COI* fragment was excluded from GenoDive analyses addressing inbreeding and HWE. To compare progeny with parents, we summarized gene diversity statistics for the *COI* sequences with the program DnaSP v.6.12.03x64 (Supplementary Table S2).

We used the R package HybridCheck v.1.0 to estimate ABBA-BABA statistics to differentiate between gene-flow versus ancestral polymorphisms. ABBA and BABA sites are parsimony-informative sites that result in discordance between gene trees and species trees due to the presence of incomplete lineage sorting or gene flow. When discordance is caused by incomplete lineage sorting, ABBA and BABA sites are expected to be equally frequent. When gene flow is present, ABBA and BABA sites have unequal frequencies. For example, an excess of the ABBA pattern indicates gene flow between the non-sister lineages P2 and P3 provided that P1 and P3 are not also exchanging genes. Patterson’s D estimate (D) is based on jackknife resampling, where statistically significant D > 0 indicates gene flow between populations P2 and P3, statistically significant D < 0 indicates gene flow between populations P1 and P3 and D = 0 indicates ancestral polymorphisms. Tests were performed on phased sequence fragments of LDH2 and PK with *B. infundibulum* s.s. as an outgroup (O) (Christianson et al., 2022).

### RADseq, shotgun, and PacBio DNA extraction and genomic sequencing for *B. microptera*

We used population genomics within *B. microptera* from the eastern Pacific to explore the evolution and connectivity of a species (Table 2). We used the E.Z.N.A. Mollusk DNA kit (Omega Bio-Tek, Norcross, GA) following the manufacturer’s protocol to extract genomic DNA from 67 specimens for restriction site-associated DNA (RAD) or whole-genome shotgun sequencing. All individuals that were sequenced for SNP calling were also Sanger-sequenced at all targeted loci (SI Supplementary Appendix A2; Supplementary Table S1). DNA was quantified using the DNA High-Sensitivity assay with the Qubit 2.0 Fluorometer (Thermo Fisher Scientific, Waltham, MA, United States). Sixteen of the individuals were used to prepare ezRADseq libraries based on modified methods (Toonen et al., 2013) fully described in (Breusing et al., 2019). As in Breusing et al., 2019, libraries were sequenced on a HiSeq 2,500 instrument at UC Davis, with a paired end 2 x 150 bp protocol. Shotgun sequencing libraries were prepared for the remainder of the 51 individuals, eighteen with a TruSeq Nano kit (Illumina Inc., San Diego, CA), and sequenced using the same protocol as the ezRAD libraries. The remainder were prepared with the Nextera XT DNA Library Preparation Kit (Illumina Inc., San Diego, CA) for lower input DNA, and sequenced on the HiSeq 4,000 instruments at UC Davis (Davis, CA) and MedGenome Labs (Foster City, CA). Four individuals were used in both ezRAD and shotgun sequencing to test for cross-platform discrepancies.

**TABLE 2:** Programs, versions, tests and parameters estimated from WGS data.

Program	Version	Tests and parameters^a^	References
ANGSD	0.935	Filter low-quality WGS data, calculate *GL, HWE, MAF, SFS, π, F*_*ST*_, admixture, and ABBA-BABA	(Li et al., 2010; Korneliussen et al., 2014)
BWA MEM	0.7.17	Map genomic reads to References sequence	Li and Durbin, (2009)
DH		*S, T*_*D*_ *, Hn, E, DHEW*	Zeng et al. (2007)
fastQC		Quality checking WGS reads	bioinformatics.babraham.ac.uk/projects/fastqc/
NGSadmix	32	Estimate individual admixture proportions from WGS data	Skotte et al. (2013)
ngsCovar		Estimate WGS correlation matrices for PCA	Fumagalli et al. (2014)
ngsDist		Compute genetic distances among individuals for MDS	Vieira et al. (2015)
ngsF	1.2.0	*F*_*IS*_	Vieira et al. (2013)
ngsTools	3.0	Package that includes ngsPopGen, ngsCovar, and ngsDist	github.com/mfumagalli/ngsTools
Pilon	11.1.5	Polish mt Genome assembly	Walker et al. (2014)
realSFS		*F*_*ST*_ , π, *W*_*θ*_ *,* heterozygosity	Nielsen et al. (2012)
SAMtools	1.7-1	WGS read formatting	Li et al. (2009)
Seqtk		Parse fastq files	github.com/lh3/seqtk
Tidyverse		Visualizing results	Wickham et al. (2019)
Trimmomatic	0.35	Trim WGS reads	Bolger et al. (2014)

a Parameters: *GL,* genotype likelihood; *HWE,* Hardy-Weinberg equilibrium; *MAF,* minor allele frequency; *SFS,* site-frequency spectrum; π, nucleotide diversity; *FST*, standardized molecular variance among populations; *TD,* Tajima’s D; *hn,* Fay and Wu’s H; *E,* Ewens-Watterson estimator; overall DHEW, compound test; *PCA,* principle component analysis; *MDS,* multiple dimensional scaling; *FIS*, individual inbreeding coefficient*;* π*,* nucleotide diversity; *W*_*Θ*_ *,* Waterson’s *Θ*.

An F3 cultured individual of *B. microptera* from the Monterey Bay Aquarium was photographed, illustrated, frozen whole in liquid nitrogen, then used to construct a PacBio CLR library, which was sequenced on a Sequel II on November 18, 2019 (D. Schultz, in prep, GenBank# JAIOUN000000000). The F3 animals in the culture were collected in December 2018. Statistical programs further detailed in Table 2.

### Sequencing and assembling the *B. microptera* mitochondrial genome

We isolated mitochondrial reads from PacBio sequencing to extract whole mitochondrial genomes from nuclear genomic data, determine gene order for *B. microptera* and use them in separate population genetic analyses from the resulting fastsq file by aligning them to the *M. leidyi* mitogenome with BWA MEM v.0.7.17. We then assembled using the Geneious v.11.1.5 assembler with standard parameters. The assembly was polished using Pilon v.1.22 with an Illumina WGS library constructed with the same DNA. The assembly was then trimmed to only include one full copy of the mitogenome.

### Separation of mitochondrial and nuclear sequences from *B. microptera*

Shotgun and ezRAD sequence data were quality checked with fastQC and trimmed with Trimmomatic v.0.35. We filtered mitochondrial data and PhiX sequencing control from nuclear loci with BWA MEM v.0.7.17, seqtk, and SAMTools v.1.7-1. Separately, nuclear or mitochondrial reads were then mapped to the PacBio nuclear or mitochondrial assemblies with BWA MEM and reformatted with seqtk and SAMtools. We aligned whole mitochondrial genomes within Geneious Prime and compared them to the *Mnemiopsis* mitochondrial genome. A minimum spanning network of mitochondrial genomes was estimated with PopArt v.1.7.

### Quality filtering whole genome sequences for *B. microptera*

To understand population connectivity and metapopulation dynamics, we ran separate analyses of mitochondrial and nuclear genomes within the program ANGSD v0.935. We removed low-quality sites for all analyses by estimating and plotting the average read depth and quality of sequencing read scores for nuclear genomes of all individuals. Visualizations enabled us to limit low quality reads, paralogs, and repetitive regions of the genome. Potentially paralogous regions were excluded by discarding reads that had multiple hits to the reference assembly and by limiting read depths from 10 to 100x coverage based on the mean read-depth distribution. SNP site mapping quality was also increased by filtering for excessive mismatches (C = 50); we removed sites with missing data, excluded spurious and improperly paired reads and computed per-base alignment qualities (baq = 1) to resolve false variants. In order to select for polymorphic sites, we set the *p* value < 1e-4 to test whether the minor allele frequency (MAF) was significantly different from zero. We tested each population for deviations from Hardy-Weinberg equilibrium (HWE) separately with the same quality filters as previous analyses (*p* value < 0.05). We calculated proportions of sites that deviated significantly from HWE within R and these sites were excluded from statistical analyses.

### Admixture and summary statistics from WGS of *B. microptera*

We used ANGSD to estimate genotype likelihoods (GL) and the MAF from counts of alleles (Li et al., 2010) to calculate the site frequency spectrum (SFS), nucleotide diversity, population differentiation (F_ST_), population structure (admixture), and inbreeding. Population genetic statistics were estimated in two ways. First, the PacBio *B. microptera* sequence was designated as ancestral, and results were “folded”. Second, reads were mapped to *M. leidyi* as the ancestor and results were “unfolded.” From those analyses we calculated summary statistics with realSFS (Table 2). Nucleotide diversity (π), Watterson’s θ, and Tajima’s D were all calculated using a 50,000-base sliding window with a step size of 10,000 bases. Per-site estimates were then calculated in R. Population admixture of individual genotypes was estimated by NGSadmix v.32 where we limited the MAF to 0.1 and ensured SNP data were present in at least 10 individuals. Plots were visualized in the tidyverse.

To test for selection and population contraction or expansion on the mitochondrial genomes, we used DH with 50,000 coalescent simulations with alpha = 0.05 for each population and *M. leidyi* (NC016117) as the outgroup to estimate Tajima’s D, Fay and Wu’s H, and the overall DHEW statistic.

### Estimating gene flow and population subdivision from WGS of *B. microptera*

We performed several tests on genomic data to estimate connectivity among populations with ngsTools and ANGSD. We calculated both weighted and unweighted estimates of gene flow (F_ST_) where unweighted is the mean of the per-site ratios and the weighted is the ratio between the sum of α and the sum of β (Reynolds et al., 1983). We used ngsCovar to estimate a correlation matrix between individuals from genotype posterior probabilities generated by ANGSD with same quality filtering parameters as above. We used the covariance matrices to estimate PCA with R function prcomp. We used ngsDist to compute genetic distances among individuals and perform multiple dimensional scaling (MDS).

To differentiate between introgression versus ancestral polymorphisms among eastern Pacific populations of *B. microptera,* we used the multi-population method within ANGSD to estimate ABBA-BABA statistics and Patterson’s D-statistic for the nuclear genomes with *M. leidyi* as an outgroup. We removed transitions and set the same filtering parameters as above from reads mapped to the assembled genome of *M. leidyi* (GCA_000226,015). We used the Rscript estAveError.R (included with the ANGSD package) to estimate Z-scores and set the limit for significance at |alpha| = 3.0.

### Heterozygosity and inbreeding coefficients from WGS of *B. microptera*

To infer whether populations were self-fertilizing or randomly mating, we used ANGSD to calculate heterozygosity and the variances of each population mean with 10 bootstrap replicates within a probabilistic framework estimated from genotype probabilities. We estimated inbreeding coefficients for each individual using the program ngsF v1.2.0. The ngsF analysis used 20 initial searches from random starting points to avoid convergence on local maxima. The run with the highest maximum likelihood was used as a starting value for the final run.

## Results

There were 425 individual ctenophores from the genus *Bolinopsis* and *Mnemiopsis leidyi* included in Sanger, ezRAD, and shotgun sequencing efforts (Table 1, Supplementary Table S2; Figure 1A, Supplementary Figure S1). The data presented in the study are deposited in the GenBank repository (accession #‘s MW786780–943, MW797317–8,170, OK086278–6,294, OK147017–7,090, PRJNA716277).

### Within and among species, genetic variability was high for the *COI* fragment

Sanger sequencing of the mitochondrial *COI* gene fragment from worldwide populations revealed four predominant lineages or clades within the genus *Bolinopsis* that were distinct at the species level, corresponding most closely to *B. infundibulum* s.s.*, B. vitrea* s.s.*, B. ashleyi* (Australia), and *B. mikado* (Japan) (Figure 1 and Table 1 and Supplementary Table S3). We provided a table of fixed differences among distinct lineages for the *COI* fragment (Supplementary Table S4). There were high levels of variability within each *Bolinopsis* lineage for the *COI* fragment, ranging from 0.1 to 3.1% Kimura two-parameter (K2P) distance (Supplementary Table S3), which was similar to other ctenophore species (Christianson et al., 2022). K2P distances between each lineage ranged from 3.6 to 22.1% (Supplementary Table S3). Distinct lineages that could not be assigned to those four described species included *B*. aff. *vitrea* from Hawaii/Moorea, an undescribed species from Australia, and two additional lineages of *B. microptera* from the west coast of North America (Figures 1A,F and Supplementary Table S3) (Christianson et al., 2022). *Bolinopsis* mitochondrial lineages were paraphyletic with *M. leidyi* and formed two well-supported sister groups: the *vitrea* complex (coral color bar, Figure 1B) and the *infundibulum* complex (gray color bar, Figure 1B (Christianson et al., 2022). Phylogenetic analyses of mitochondrial *COI* data combined with *28S* and *H3* nuclear fragments also supported these clades (Figure 1B). Sequence data from two nuclear gene fragments (*PK* and *LDH2*) supported that *B. microptera* from the eastern Pacific Ocean were distinct from *B. infundibulum* s.s. The nuclear fragment for *H3* contained shared ancestral polymorphisms between the three lineages, while attempts to amplify *LDH1* and *MDH* were unsuccessful for *B. infundibulum* s.s. (Supplementary Figure S1).

We sequenced 191 *Bolinopsis microptera* specimens for *COI* from the two Eastern Pacific lineages collected from surface waters to ∼2,000 m depth and as far as 160 km offshore of western North America. The two lineages differed by 3.6% K2P for the *COI* fragment. One lineage occurred from Friday Harbor, WA to the Santa Lucia Knoll offshore of Point Conception, CA, and the other was found from the Monterey Bay area to the Channel Islands, CA. We subdivided lineages by three collection sites, designated as WA (Friday Harbor, WA), NorCal (Monterey Bay area, CA), and SoCal (San Luis Obispo–Channel Islands, CA). The highest levels of mitochondrial diversity were found in California populations, in part because they included both the California and the Washington mitochondrial lineages (Supplementary Table S3; Figure 1F, Supplementary Figure S1).

### Morphological differences among *Bolinopsis* species are subtle

We did not see dramatic differences among five of the *Bolinopsis* species, and the differences in ages of specimens contributed to variability since body proportions change over time. Boxplots of each measurement showed there were high levels of within-lineage variability and that *M. leidyi* was the most divergent (Supplementary Figure S2). However, the clades we recovered from molecular data (Figure 1B) were concordant with subtle differences in morphology. The tSNE plot based on combined measurements showed two clusters including the *vitrea* and *infundibulum* complexes (Figure 1E).

**FIGURE 2:**
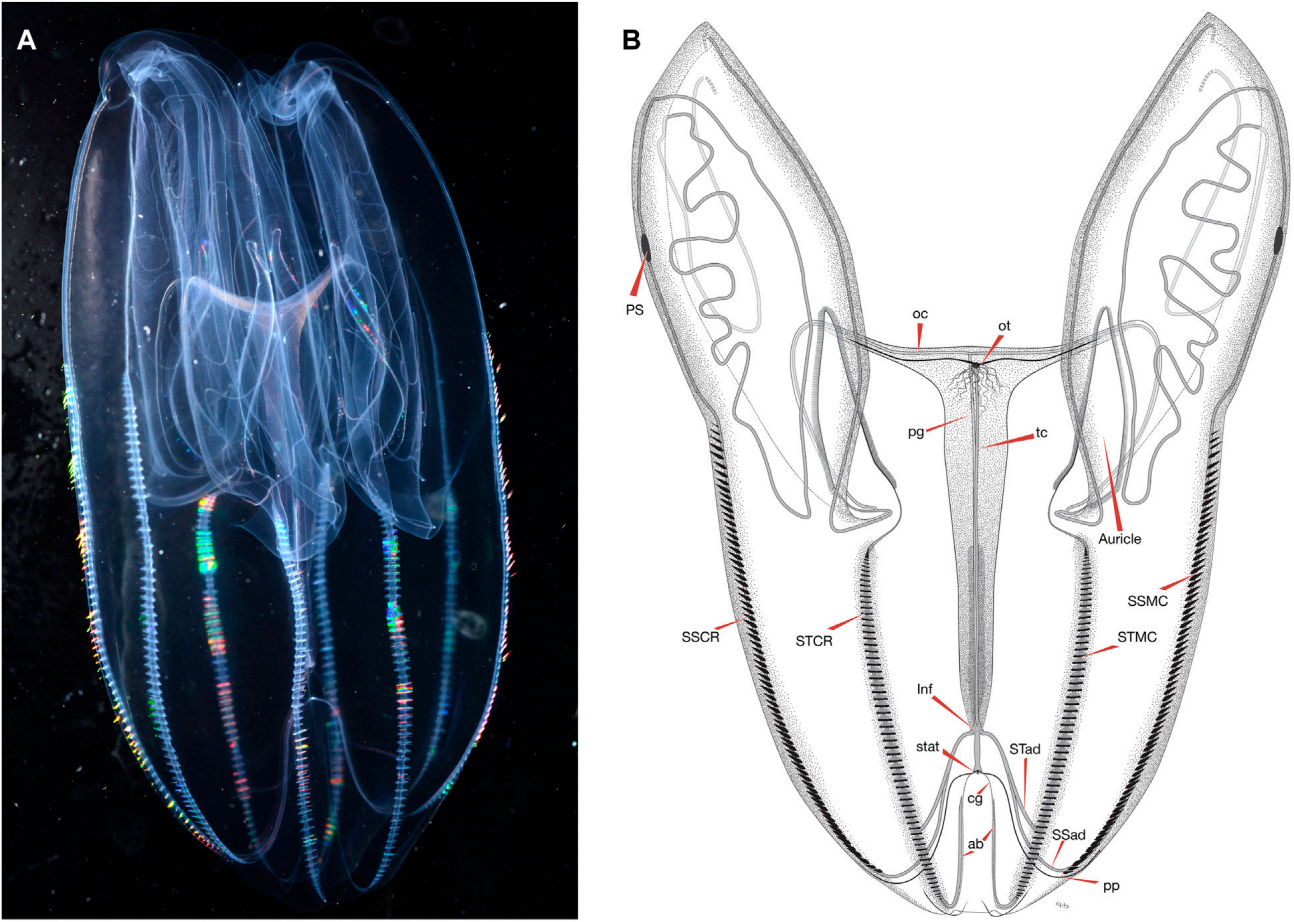
**(A)** An image of *B. microptera* from eastern Pacific Ocean with a “relaxed” body position and **(B)** an illustration of *B. microptera* from the eastern Pacific Ocean with morphological landmarks indicated by arrows for: auricle, oral tentacle (ot), pigment spot (PS), oral canal (oc), paragastric canal (pg), tentacular canal (tc), sub-stomodaeal comb row (SSCR), sub-tentacular comb row (STCR), infundibulum (Inf), ciliary groove (cg), aboral projection (ab), sub-stomodaeal meridional canal (SSMC), sub-tentacular meridional canal (STMC), sub-tentacular adradial canal (STad), sub-stomodaeal adradial canal (SSad), and the pole plate (pp). Image and illustration are representative of full-grown specimens, body proportions change as the species mature.

One morphological feature that differs between *B. microptera* and *B. infundibulum* s.s. is the way that the meridional canals connect at their aboral ends to the infundibulum (the canal-hub at the base of the stomodaeum). In both species, the eight meridional canals are fed near their aboral ends by four interradial canals arising from the infundibulum, each of which bifurcates once into eight paired adradial canals. In *B. infundibulum* s.s., all eight adradial canals connect directly to the extreme aboral end of the meridional canals (Mayer, 1912, Plate 4). In *B. microptera* (Figure 2), the canals are connected in two different ways: while the branches that feed each substomodaeal meridional canal (ssmc) merge with that ssmc at its aboral end (as in *B. infundibulum* s.s*.*), the four branches that feed the subtentacular meridional canals (stmc) meet the stmc somewhere between the aboral tips of the comb rows to the level of about the 30th comb plate above the aboral ends of the comb rows. The position of this connection point varies between animals—likely associated with overall size—but it is consistent within each animal. At the aboral end of each comb row, below the junction with the adradial canal, each stmc then narrows, curves upward towards the statocyst, and ends blindly. The blind extension of the meridional likely sustains the ciliary groove that is adjacent to it, in the same way that the meridional canals provide sustenance for the comb rows. In one specimen from California, the left canal projection was very slightly wider than the right (Figure 2), but this was not seen in the Friday Harbor specimens.

We also examined morphological distinctions between populations of *B. microptera* with the same measurements and while there was variability within populations, we found no consistent differences (Supplementary Figure S3).

### Parameters of the *B. microptera* mitochondrial genome

The *B. microptera* mitogenome length was 10,476 bp, which is similar to the size of previously reported ctenophore mitogenomes. The base composition of the forward strand was 21.3% A, 11.4% C, 8.7% G, 58.6% T, and 20.1% G + C. The gene content was the same as that of *M. leidyi* and other reported ctenophore mitogenomes, although the gene order (COX1-COX3-12S-16S-ND4-ND2-ND4L-ND5-ND1-ND3-COX2-CYTB-ND6) was different from the *M. leidyi* mitogenome gene order (COX1-COX3-ND3-COX2-12S-16S-ND4-ND2-ND4L-ND5-ND1-CYTB-ND6) despite being a closely related species. The *B. microptera* mitogenome also contains one unidentified reading frame (URF) that appears to encode a 55-amino acid peptide. All 12 *B. microptera* mitogenome ORFs used the TAA termination codon.

### Whole mitochondrial genome data mirrored *COI* patterns for *B. microptera*

**FIGURE 3:**
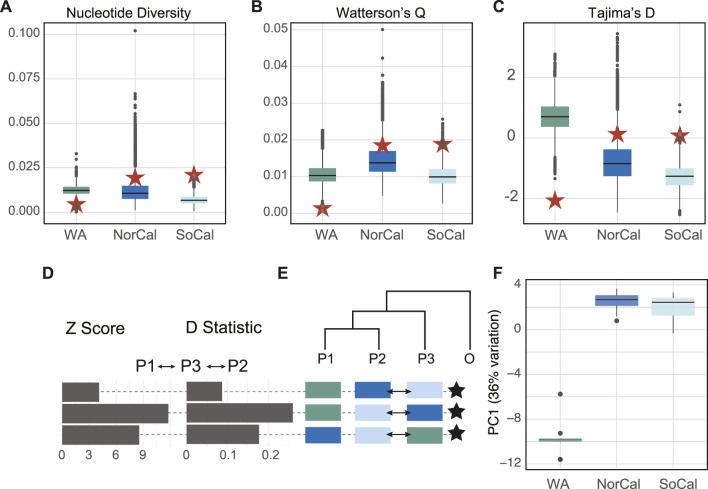
Per-site estimates of **(A)** nucleotide diversity (π), **(B)** Waterson’s θ, **(C)** Tajima’s D test statistics for nuclear (box plots) and mitochondrial genomic data (red stars), **(D)** Z-scores and D-statistics for ABBA-BABA comparisons between populations with *M. leidyi* as an outgroup for nuclear genomes, **(E)** Cartoon tree represents hypotheses to test whether population 3 (P3) shared more ancestral versus derived alleles with population 1 (P1) relative to population 2 (P2). Positive-shifted histograms (excess of ABBA) represent significant gene flow between P2 and P3, negative-shifted histograms (excess of BABA) for significant gene flow between P1 and P3. Arrows on tree cartoons indicate gene flow between populations. Stars indicate significant z-scores > three standard deviations from zero. **(F)** Box plots of the first PCA axis of nuclear genomes for each population mapped to *M. leidyi* for 14,396 polymorphic sites that represented 36% of variation. Populations colored by locality: Friday Harbor (WA, green), the Monterey Bay area (NorCal, blue) and San Luis Obispo–Channel Islands, CA (SoCal, light blue).

We analyzed a total of 67 complete mitochondrial genomes from the two lineages of *B. microptera* from the eastern Pacific Ocean. Network topologies and within/among population divergence of whole mitochondrial genomes and the *COI* fragment showed the same pattern, where the WA mitochondrial lineage was divergent (K2P∼2%) but sympatric with CA populations (Supplementary Table S3 and Supplementary Figure S1). Folded SFS (site frequency spectrum) plots showed the mitochondrial genomes of the WA population had the highest number of singleton SNPs and the lowest levels of variability compared to the CA populations (Supplementary Figure S4A; Supplementary Table S5). Mitochondrial genomes from NorCal and SoCal populations had the highest number of segregating sites and an order of magnitude greater diversity indices compared to WA (red stars on Figure 3; Supplementary Table S5). Both the Tajima’s D and the DHEW compound statistic indicated that the WA population was under selection and California populations were neutral (Supplementary Table S5).

### *Bolinopsis microptera* populations displayed cytonuclear discordance

**FIGURE 4:**
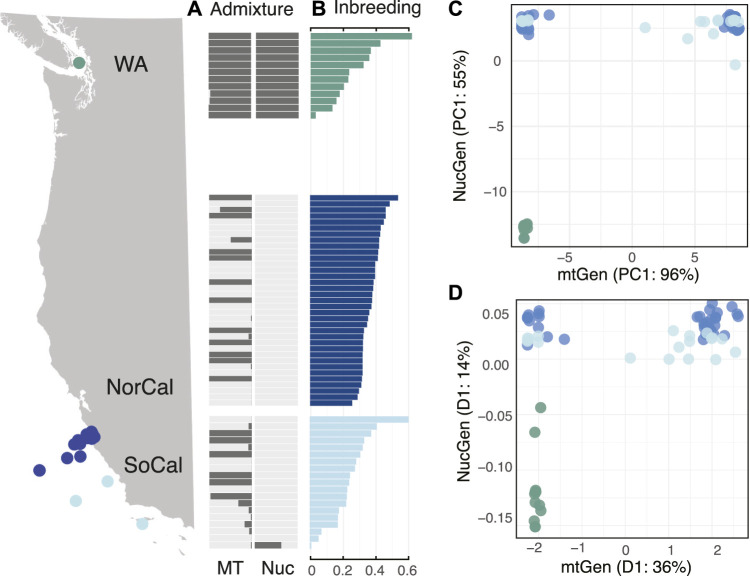
Population genomic results of the three populations including WA (green), NorCal (blue), and SoCal (light blue) for **(A)** NGSadmix results for mitochondrial and nuclear genomic assignment tests for two populations, sorted by **(B)** per-individual inbreeding coefficients, **(C)** first principal component from PCA estimates of nucleotide diversity for mitochondrial (x-axis) versus nuclear (y-axis) genomes, and **(D)** first dimensions of MDS of genetic distances for mitochondrial (x-axis) versus nuclear (y-axis) genomes.

Nuclear genomic data included 1,583,318 shared single nucleotide polymorphisms (SNPs) and showed different patterns of population subdivision than mitochondrial data. The WA mitochondrial lineage was also present in CA populations but the WA nuclear lineage was restricted to Washington. Admixture plots of nuclear versus mitochondrial data clearly illustrated cytonuclear discordance in California populations where the WA mitochondrial lineage was admixed throughout California but with one exception the WA nuclear lineage was restricted to Washington (Figure 4A, Supplementary Figure S5A).

The nuclear genomes were less variable than the mitochondrial genomes of *B. microptera* and were the most similar within California, regardless of whether the animal had a WA mitotype. We detected one individual that was a putative WA/CA hybrid from the SoCal population (Figure 4, Supplementary Figure S5A).

We also examined discordance by plotting the first dimension of covariance matrices for principal component analyses (PCA) and distance matrices for multidimensional scaling (MDS) of mitochondrial versus nuclear genomes (Figures 4C,D). PCA plots showed three distinct clusters including WA_nuclear + mito_, CA_nuclear_ + WA_mito_ and CA_nuclear_ + CA_mito_. The putative “hybrid” individual from SoCal was intermediate to the CA populations. (Figure 4C). MDS corroborated cytonuclear discordance by recovering the same three clusters as PCA (Figure 4D).

Sanger-sequenced data also showed the same pattern of discordance where the WA mitochondrial lineage was found throughout California but the WA nuclear lineage was not (Supplementary Figures S1, S5B).

Levels of nucleotide (π) and genetic diversity (Watterson’s θ) were similar in the nuclear genomes of all three populations (Figure 3), but conversely, lowest for the WA population among the mitochondrial genomes. The mean Tajima’s D statistic for nuclear genomes was highest for the WA population (*TD* = 0.711) indicating a lack of rare alleles and possible population contraction, while for the CA populations it was slightly negative (NorCal *TD* = -0.781 and SoCal *TD* = −1.284) indicating an abundance of rare alleles and possible recent expansion. These results also were opposite of what we found for mitochondrial genomes (Figure 3; Supplementary Table S5). Per-individual inbreeding coefficients varied within and among populations and were relatively high (0.01–0.62) (Figure 4B).

### Gene flow analyses showed isolation among Eastern Pacific *Bolinopsis* populations

We analyzed gene flow and demographic histories among the three Eastern Pacific populations. Pairwise FST values estimated using both allele frequencies from Sanger and WGS nuclear data showed significant differentiation between WA and CA populations, but not within CA (Supplementary Table S6). Directional migration patterns, effective population sizes, and time of population splitting were estimated by running IMa3 on Sanger-sequenced nuclear fragments and mitochondrial genomes. IMa3 analyses revealed significant bidirectional migration between NorCal and SoCal, and lower levels from SoCal into WA populations. The Washington population was isolated from the NorCal population. There was also significant immigration from an unsampled population, which was unsurprising given the limits of our collections. The effective population sizes (θμ) were highest for SoCal and NorCal populations, reflecting high levels of diversity, especially for mitochondrial data. The estimated date that the population split (τμ) was relatively recent (Supplementary Table S7). BA3 analyses based on allele frequencies had similar results, where there was gene flow between NorCal and SoCal while WA was mostly self-seeded (Supplementary Table S8).

We used ABBA-BABA tests (D-statistics) to differentiate between incomplete lineage sorting (ancestral polymorphisms) and introgression (ongoing gene flow) between the populations for 14,396 sites in the nuclear genomes, with *M. leidyi* as an outgroup. These tests showed significant introgression between NorCal and SoCal and also between WA and SoCal. We performed PCA with the same data to examine population structure and found that the first PC explained 36% and the second only 1.5% of variation between populations. While the WA population was the most distinct, SoCal was slightly more similar, surprisingly, to WA than to NorCal (Figure 4).

### Crossing experiment revealed asymmetrical reproduction

We performed crosses of WA and CA *Bolinopsis* at the Monterey Bay Aquarium, resulting in many thousands of progeny (WA×CA). We collected two different batches of ∼100 progeny 1 week apart for sequencing analyses. All of the ∼200 progeny sequenced for *COI* had an identical mitotype (data not shown). We used a number of tests on the progeny and putative parents to determine whether the progeny represented “hybrids”: (1) whether multiple parents contributed to crosses, (2) whether there was asymmetrical reproduction, and (3) whether populations were randomly mating. Because all progeny sequences were identical for the *COI* fragment, it initially appeared either that one individual self-fertilized or one ‘mother’ spawned. However, sequences of the four nuclear Sanger loci were variable among the progeny and revealed several heterozygous positions for all markers, indicating that at least two parents contributed to genetic diversity (Supplementary Figure S3). Multi-locus tests showed that WA and NorCal populations varied significantly from Hardy-Weinberg Equilibrium (HWE) (*p* ≤ 0.001), but the SoCal and WA×CA populations did not. The hybrid index results showed all but one of the progeny had a higher affinity to the NorCal population. We used ABBA-BABA tests to distinguish gene flow (introgression) from ancestral polymorphism in the two polymorphic markers for which an outgroup was sequenced (*LDH2* and *PK*). ABBA-BABA tests were not significant for *LDH2* but showed that for the *PK* fragment, the highest probability of gene flow was between CA and the progeny (Bold values, Supplementary Table S9).

## Discussion

### Speciation in open water, gene flow despite barriers

Ctenophores have high rates of mitochondrial evolution and reduced, derived mitochondrial genomes compared to many other animal phyla (Pett et al., 2011; Kohn et al., 2012; Schultz et al., 2020). We found that *B. infundibulum* s.s. had a broad pan-Arctic distribution despite many physical barriers to gene flow but conversely, that the “*vitrea* complex” was represented by many distinct lineages despite few apparent barriers to gene flow. Our findings were in contrast to the idea that oceanic ctenophores are cosmopolitan and that they are limited to one mode of speciation, even within a genus. There are many processes that are thought to drive the evolution of species, most of which are studied in terrestrial or benthic model marine populations, and these habitats usually include clear boundaries to reproduction. The pelagic environment offers few clear and easily measurable boundaries. Perhaps rapid mitochondrial evolution, self-fertilization, and distinctive physiological adaptations found in *Bolinopsis* contributed to the incongruous patterns of speciation we found within the genus.

### Mitochondrial divergence in *B. microptera*: Two species or one?

We found two divergent mitochondrial lineages of *Bolinopsis microptera* along the eastern Pacific coast that differed by ∼3% K2P and were sympatric in California. Although this level of divergence within a species is relatively high, it was similar to within-species diversity of other ctenophores, including *B. infundibulum* s.s. (Christianson et al., 2022). Concordant nuclear loci are critical to accurately delimit among species (Avise and Ball, 1990) and while we found the Washington population was isolated from California populations (Supplementary Tables S3–S6), nuclear data were much less variable than mitochondrial data (Figure 4, Supplementary Figure S1) and the corresponding gene trees did not reproduce an organismal phylogeny (Figure 1B). We concluded that Eastern Pacific *Bolinopsis* represented one species from Washington state to California.

### One distinct MOTU that has a name

Most ctenophore names have many historic synonyms and *Bolinopsis* is no exception (Mills, 2020). Based on work by (Mortensen, 1912), nine species described from 1780 to 1888 have been synonymized under *B. infundibulum* alone (Mills, 2020). In the North Pacific, *Bolina septentrionalis* Mertens (1833) was described from the shores of St. Matthew (“Matthaei”) Island in the Bering Sea by Mertens and identified in the Gulf of Georgia (Washington Territory) by A. Agassiz (Agassiz, 1860). A few years later, Alexander Agassiz re-examined his drawings and descriptions of Washington specimens from Rosario Strait (Washington State) about 20 miles from and contiguous with both Friday Harbor and the Gulf of Georgia (now called the Strait of Georgia). He concluded that *Bolinopsis* ctenophores from the western coast of North America were distinct from Mertens’ arctic *B. septentrionalis* because they were more elongated, had shorter lateral lobes, and had more complicated “windings” of the canals in the lobes (“ambulacral tubes”). The new species was designated as *Bolina microptera* (Agassiz, 1865). The genus name *Bolina* was already occupied by a mollusc, so it was eventually changed to *Bolinopsis* (Mayer, 1912). *Bolinopsis septentrionalis* was synonymized with *B. infundibulum* by (Mayer, 1912). Mortensen, (1912) concurred that *B. septentrionalis* is a synonym of *B. infundibulum* and also noted substantial morphological variation in the *B. infundibulum* specimens that he studied in Trondheim, Norway in the summer of 1911. He thus synonymized *B. micropter*a of A. Agassiz with *B. infundibulum* as being insufficiently different, noting that “the question can perhaps not be regarded as definitely settled at present.” In her 1966 PhD thesis, Helen L. Dunlap (Dunlap, 1966) recognized that the *Bolinopsis* she studied in Friday Harbor, WA represented a distinct species and was probably *Bolina microptera* of Agassiz, 1865 based on location, although she stated ‘a more complete description is in order’. Morphologically we found a discrete feature which appears to differentiate *B. microptera* from *B. infundibulum* s.s.: In *B. infundibulum* the four subtentacular meridional canals (Figure 2: stmc) connect to the adradial canals at their distal-most aboral end, while in *B. microptera* the subtentacular adradials make a nearly perpendicular connection to the subtentacular meridional canals somewhere a short distance above or just below the aboral section of comb plates. Aboral to the connection point, the stmcs end in blind extensions (Figure 2: ab). This is discussed more in the description contained in Supplementary Material SA1. Although the original species description lacks an illustration, we reinstate the name *Bolinopsis microptera* (A. Agassiz, 1865) for *Bolinopsis* ctenophores that inhabit eastern Pacific waters including Washington to California based on genetics, geography and morphology (Supplementary Table S3; Figures 1, 2, Supplementary Figures S1–S3); this is the only species of *Bolinopsis* found in these waters. We thus refer to *B.* aff. *infundibulum* from the eastern temperate North Pacific as *B. microptera*.

### Cytonuclear discordance and metapopulation dynamics in *B. microptera*

Although we found little evidence to support the presence of two distinct species of *Bolinopsis* in the Eastern Pacific, *B. microptera* had high levels of within-species variation and occupied a small range compared to *B. infundibulum* s.s. Most interestingly perhaps, our data revealed that the distribution of nuclear genotypes was discordant with that of mitotypes off the California coast (Figure 4, Supplementary Figure S5). Typically, the high level of differentiation we saw in *B. microptera* is characteristic of allopatric populations that have undergone long periods of isolation, differing levels of selection, and/or high levels of genetic drift (Coyne and Orr, 2004). Many scenarios can lead to highly divergent sympatric mitochondrial lineages, such as mitochondrial pseudogenes inserted into the nuclear genome (numts) (Benasson et al., 2001), the presence of cryptic species, hybridization or some other driver of a non-random mating, or contact between long-isolated populations. We ruled out the presence of numts since we did not observe frameshifts, stop codons, or double peaks in mitochondrial sequence data. We also ruled out the presence of cryptic species based on the low amount of differentiation of nuclear genomic data among populations. In the following sections, we explore other possible causes of discordance: hybridization via secondary contact after geographical isolation, selection, and asexual reproduction.

### Cytonuclear discordance because of hybridization

Discordance between mitochondrial and nuclear genomes is often detected in hybrid zones (Arnold, 1993) as a result of non-random mating due to natural selection and/or demographic asymmetries (Toews and Brelsford, 2012). One other population genetic study focused on ctenophores also found cytonuclear discordance between a mitochondrial fragment and a panel of nuclear microsatellite loci for the neritic species *Mnemiopsis leidyi* (Bayha et al., 2014). In that case, the pattern of discordance is due to differences in evolutionary rates between microsatellites and divergent allopatric mitochondrial lineages (Bayha et al., 2014), a phenomenon commonly observed when comparing different markers (Toews and Brelsford, 2012). Conversely, our work showed that California populations of *B. microptera* exhibited a significant pattern of cytonuclear discordance, where the WA mitotype was frequent, but the nuclear genotype was absent (Figure 4, Supplementary Figure S5).

Hybrid zones occur when distinct populations meet, mate, and produce progeny. Hybridization between distinct populations or species can occur for a number of reasons, whether they are exogenous (due to the environment) or endogenous (due to genetic differentiation) (Barton and Hewett, 1985; Arnold, 1997). Two hypotheses of intergradation include (1) primary: a contact zone between distinct habitats or steep environmental gradients that result in parapatric speciation, or (2) secondary: resulting from contact between previously allopatric populations (Barton and Hewett, 1985). *Bolinopsis* ctenophores include common and abundant oceanic species that live from the surface to nearly 2,000 m depth; it is therefore unlikely the population subdivision we detected was due to steep environmental gradients that would drive parapatric speciation in open ocean waters. It is plausible however, that sea-level changes since the last glacial cycle isolated populations that inhabited protected coastal embayments such as the Gulf/Strait of Georgia and Puget Sound, WA (Lambeck, 2004). Patterns of cytonuclear discordance in California and high levels of inbreeding (Figure 4, Supplementary Figure S5) detected herein suggested that California *Bolinopsis* engaged in secondary contact between two long-separated entities. These findings were unusual since hybrid zones are often characterized by a somewhat narrow cline. The pattern we uncovered included a large swath of California (and possibly Oregon and Washington state) that was in discordance.

As the presence of the WA genotype in CA was rare, it is plausible that the Washington mitotype invaded the California populations via adaptive introgression, or that cytonuclear incompatibilities existed between California mitotypes and Washington genotypes. We examined a number of hypotheses that could explain the patterns we saw.

### Isolation maintained by selection and local adaptation

Reproductive isolation can be driven by a few individuals that colonize and rapidly expand to fill a new area. Selection and drift in the new population cause a rapid shift to a new co-adapted combination of alleles (Barton, 1989). Local adaptation can lead to phylogeographic structure since there are a number of proteins encoded in the genome that are exported to mitochondria and thus interact with proteins encoded in the mitogenome, resulting in “cytonuclear coevolution.” Thus, local adaptation might cause nuclear gene sequences to diverge, which could likewise promote divergence in the mitochondrial genomes. Smaller populations, especially those with high FST values, are more subject to random drift since mutations have bigger effects (Whitlock, 2004). High fecundity and high rates of mitochondrial evolution in *Bolinopsis* ctenophores could accelerate these processes. Our data indicated the presence of selection on mitochondria in the Washington population, which had the smallest effective population size. Tajima’s D values for the WA mitochondrial genomes also were negative; while rare alleles were abundant, this can be interpreted as evidence for a recent selective sweep or population expansion after a bottleneck. We also found that all of the populations deviated from Hardy-Weinberg expectations and were therefore not randomly mating. It is plausible that sea level changes over time historically isolated the Washington population in a neritic rather than oceanic environment, thereby contributing to patterns of selection, genetic drift, and local adaptation.

Another common mechanism of reproductive isolation is asynchronous spawning, which could perpetuate divergence between Washington and California populations despite a lack of obvious contemporary geographic boundaries. In our laboratory experiment, Washington and California adults spawned asynchronously. In the Monterey Bay area, *Bolinopsis* ctenophores are abundant in the summer, and are present but rare in late fall and winter months (Figure 1C), whereas in Washington, they are most abundant in the spring (C.E. Mills *pers. obs.*), but present in low numbers throughout the year. We did not observe spatial or temporal segregation between the two mitotypes collected in the Monterey Bay area, further indicating a well-mixed population. However, divergent reproductive histories (i.e., one population spawning earlier in the year or day) could be a remnant of local adaptation in northern populations despite evidence of connectivity in pelagic habitats.

Natural selection has greater influence on systems with larger population sizes and species with large populations may be particularly likely to exhibit selection-driven phylogeographic structure (Irwin, 2012). The effective population sizes from California were an order of magnitude larger than Washington populations Supplementary Table S7), a pattern mostly driven by high levels of mitochondrial variation. Selection on mitochondrial loci can also result in cytonuclear discordance (Irwin, 2012). Tajima’s D values were neutral for mitogenomic data from both California populations, but negative for their nuclear genomes, consistent with a recent selective sweep or bottleneck. The low levels of heterozygosity we observed in California populations could also indicate high levels of inbreeding and/or self-fertilization.

### Non-random mating: Self- versus cross-fertilization

Inbreeding due to self-fertilization can have effects on genome evolution, as it reduces effective population size, limits gene flow via gamete migration, and reduces the effective recombination rate between polymorphic sites. However, it is unlikely to have much effect on uniparentally inherited mitochondrial genes. Patterns of discordance can be caused by genetic drift, which has greater influence on systems with smaller population sizes (Irwin, 2012) since inbreeding reduces the effective population by limiting the total number of alleles an individual has (Whitlock, 2004). Most ctenophores, including *Bolinopsis*, are self-fertile simultaneous hermaphrodites, begin adult reproduction relatively young, spawn continuously (Edgar et al., 2022), and have the ability to produce thousands of eggs a day that then undergo rapid planktotrophic development (Strathmann, 1987). In individual *Bolinopsis,* sperm release occurs for 5 minutes in several bouts and precedes oocyte release. Nearly all *B. microptera* from Washington spawn about 1.5–2 h after exposure to light, so reproduction is seemingly synchronized among individuals. However, with the eggs released immediately after the sperm, there is a high probability of self-fertilization (Dunlap-Pianka, 1974). In addition, some ctenophores are able to reproduce sexually while they are still larvae (dissogeny) (Martindale, 1987). The pattern of rapid reproduction and massive die-offs can create multiple bottlenecks which causes mitochondrial and nuclear genes to accumulate deleterious mutations. This pattern could be amplified by individuals that self-fertilize. Pett (2011) showed, “A small effective population size can facilitate stochastic fixation of deleterious mutations in genes responsible for DNA replication and repair that can lead to an increased mutation rate.” High levels of self-fertilization in ctenophores, which are known to have accelerated rates of mitochondrial evolution, could intensify genetic drift and thereby population differentiation.

## Conclusion

Our results revealed cryptic drivers of genetic isolation and population subdivision, which stand in stark contrast to the cosmopolitan model traditionally applied to pelagic species. *Bolinopsis* ctenophores are abundant, oceanic species that occupy a large depth range and are likely able to overcome physical barriers. For example, *B. infundibulum* had a circumpolar distribution despite large land masses within its range. However, we also found seven distinct lineages of *Bolinopsis* that mostly partitioned geographically throughout the world ocean. Large distances, the incredible complexity of ocean circulation patterns, and physiological adaptations (or limitations) must have had significant effects on the patterns of local retention, gene flow, and speciation of *Bolinopsis*. We found California represented a massive zone of cytonuclear discordance in *B. microptera,* a pattern seldom detected in open populations. Geography and historical sea-level changes may have contributed to isolation of the WA population of *B. microptera*. The dock at Friday Harbor Laboratories, where we collected Washington ctenophores, is in a protected bay in the Salish Sea, over 100 km from open Pacific coastal waters. Isolation, combined with accelerated rates of mitochondrial evolution, small effective population sizes, and selection, could have driven population differentiation on a relatively fast timescale. Additional collections from the waters offshore of Washington state would be informative and would help to ascertain whether discordance was due to inbreeding (selfing), reproductive isolation (asymmetrical spawning), complex local oceanic circulation patterns, or some combination of the above. Future studies of ctenophore population genetics can illuminate the evolution and biodiversity of this rarely studied group and help to understand worldwide population subdivision in what were thought to be mostly cosmopolitan species.

## Data Availability

The data presented in the study are deposited in the GenBank repository, accession numbers: MW786780-MW786943, MW797317-MW798170, OK086278–6294, and OK147017–7090 https://www.ncbi.nlm.nih.gov/genbank/, PRJNA716277.
