# Supplementary figures and images for: Speciation of pelagic zooplankton: Invisible boundaries can drive isolation of oceanic ctenophores
Source: Front Genet. 2022 Oct 7;13:970314. doi: 10.3389/fgene.2022.970314 (PMC9585324; doi:10.3389/fgene.2022.970314)

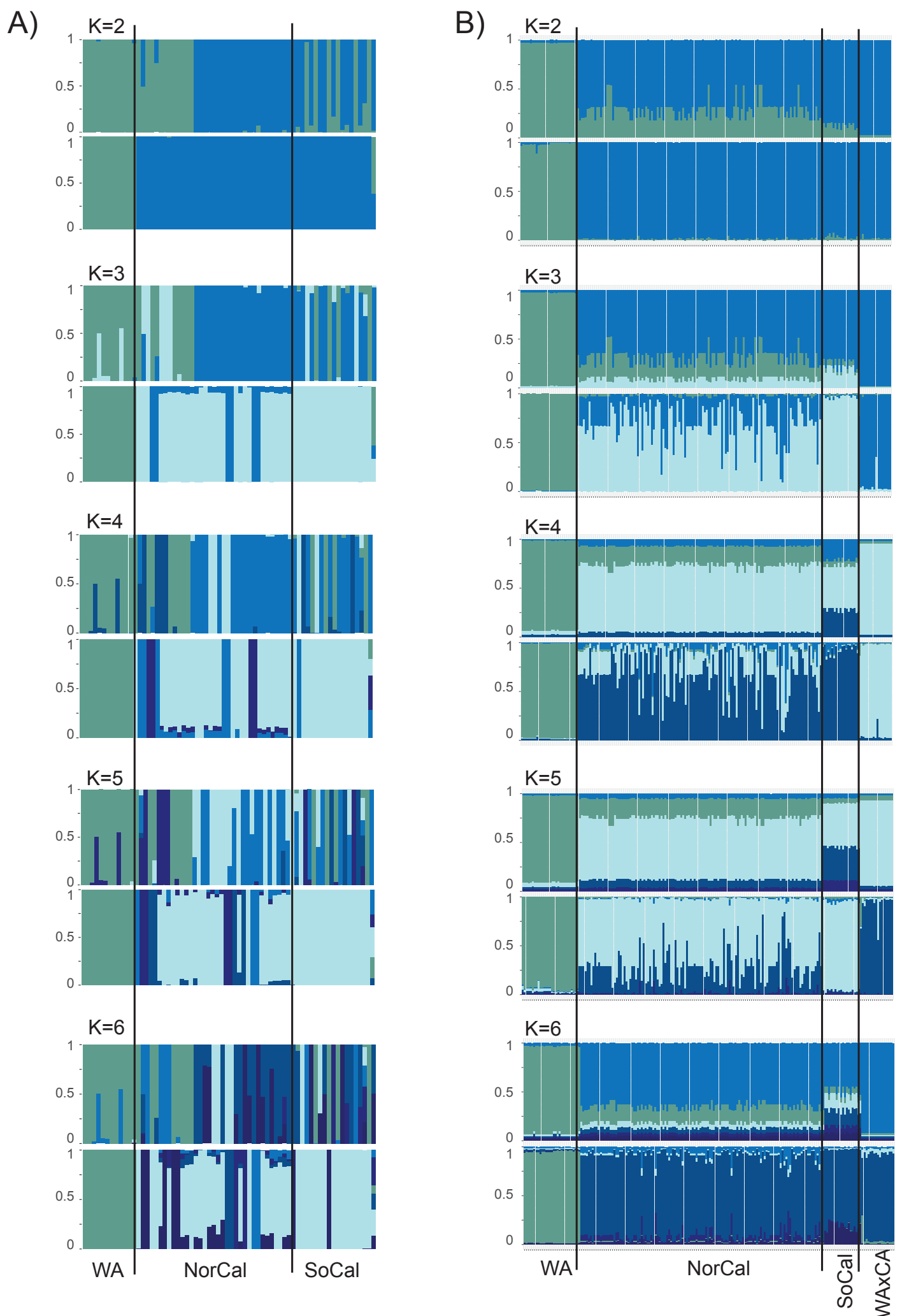

Supplement: Supplementary file 1 [file Image5.pdf]
