# Supplementary material for: Speciation of pelagic zooplankton: Invisible boundaries can drive isolation of oceanic ctenophores
Source: Front Genet. 2022 Oct 7;13:970314. doi: 10.3389/fgene.2022.970314 (PMC9585324; doi:10.3389/fgene.2022.970314)
Supplement: Supplementary file 2 [file Table1.docx]

***Supplementary Material***

**1 Supplementary Data**

**Appendix 1. Redescription of *Bolinopsis microptera* A. Agassiz 1865**

*Diagnosis*. Lobate ctenophores with a pair of large oral lobes approximately half of the overall length of the animal, that are not provided with swimming muscles. Usually lying just inside the lobes are two pairs of flat, largely triangular, lanceolate auricles, about 1/3 the length of the lobes. The substomodaeal meridional canals (MCs) run straight up beyond the comb rows tracing the outline of the lobes and filling the middle area of the lobes with 8–10 half loops on each side before meeting near the midline. At the aboral ends of the meridional canals, adradial canals (Figure 2: SSad) join the **substomodaeal** meridional canals at the aboral extreme end of the comb rows, with the adradials running parallel to the course of the MCs, ultimately forming complete loops. In contrast, the adradial canals tee into the **subtentacular** meridional canals some distance above the aboral ends of the comb rows (Figure 2: STad). Below the connection point the four subtentacular meridional canals narrow, curve upward towards the statocyst, and end blindly (Fig 2B, ab).

*Description*.

Body and Oral Lobes. Body to at least 100 mm in total length, moderately compressed in the stomodaeal axis; the overall body proportions are somewhat variable within a general construct. The expanded lobes are approximately as long again as the rest of the body, but can be a little more or less than half the overall length. The consistency of this animal is soft and flexible, but in general it can survive being gently poured out of collection containers and remain intact. It is very transparent and colorless, except that some specimens have black pigment in several spots (PS, Figure 2B) or a continuous black to deep purple line may occur along the parts of the substomodaeal meridional canals that run up the outside of the lobes.

Comb Rows. The four substomodaeal comb rows are somewhat longer than the four subtentacular comb rows, extending orally a short distance onto the lobes and are thus a little longer than half the total body length. The subtentacular comb rows (STCRs) extend slightly deeper aborally than the substomodaeal comb rows, on gelatinous extensions of the body at the aboral end that cause the aboral organ (Figure 2; stat) to be deeply sunken. The STCRs extend orally less than half of the total body length, each ending at a small, unciliated gelatinous tab, with long cilia then continuing packed in a single row along the edge of a flat, largely triangular, lanceolate, auricle that lies inside the lobe and is about 1/3 as tall. A large specimen might have 50–60 comb plates in each comb row.

Statocyst and Pole Plates. The statocyst is deeply sunken at the aboral end, in a slightly flattened, conical cavity formed by the aboral projections of the body, lying about 1/4 of the distance from the aboral end to the mouth. The pole plates are more or less straight and narrow, extending out onto the sides of the body between the substomodaeal comb rows about as high as several comb plates up from the aboral end.

Tentacular Apparatus. In adult specimens, the tentacular apparatus is usually a small whitish round remnant served by the tentacular canal, with a cluster of short tentilla, but in one specimen that was nearly 50 mm long, a short tentacle with tentilla was still evident emerging from the tentacle bulb.

Gastrovascular System. The mouth is located above and between the bases of the lobes, opening out into an unpigmented stomodaeum; the edge of the mouth and the surface of the stomodaeum are smooth. The infundibular canal below the stomodaeum is very short, only about ¼ as long as the statocyst is deep in its cavity. A pair of tentacular canals arise from the small subpharyngeal infundibulum at the same level as the pair of narrow and smooth (without diverticula) paragastric canals, almost immediately overlying the paragastric canals; all four run up the midline of the stomodaeum in the stomodaeal plane with the paragastric canals bifurcating out along the edge of the mouth and the tentacular canals ending just below this level at the remnant tentacle bulbs. Four interradial canals also leave the infundibulum at the same level as the paragastric and tentacular canals, but point downward at an angle, each bifurcating to form eight adradial canals. The four adradial canals that feed the four substomodaeal meridional canals (Figure 2: SSad) merge with those canals at the aboral end of the comb rows, ultimately forming complete loops. In contrast, the four adradial canals that feed the four subtentacular meridional canals (Figure 2: STad) merge nearly perpendicularly with those canals some distance above the aboral ends of the comb rows (somewhere from the aboral tips of the comb rows to the level of about 1/4 of the length above the aboral ends of the comb rows, varying between animals, but consistent within each animal). This connection of the adradial canals to the STMCs appears to be a feature that distinguishes *B. microptera* from *B. infundibulum*; in *B. infundibulum*, both the subtentacular and substomodaeal adradial canals are depicted as joining at the extreme aboral end of the meridional canals, and parallel or tangent to the MCs. In the aboral direction, below the junction with the adradial canal, each subtentacular meridional canal then narrows, curves upward towards the statocyst, and ends blindly. Orally, the substomodaeal meridional canals continue straight up beyond the substomodaeal comb rows, nearly to the top of the lobes, where they turn and follow the outline of the lobes in both directions down to the level of the bases of the auricles, then winding in a series of up to 8–10 half-loops running around the inside surface of the oral lobes back to the top and then dipping down again to about the center where they fuse near the midline. The subtentacular meridional canals run up orally along the edges of the auricle, and then follow the edges of the lobes, where they fuse near the midline. The oral branches of the paragastric canals do not ring the mouth, but at the ends of the mouth, they each bend down and eventually fuse with the subtentacular meridional canals at the base of each auricle, where the STMCs then move out onto the lobes.

Gonads and Developmental Stages. Gonads develop all along the walls of the meridional canals beneath the comb plates, with ovaries producing eggs on the perradial sides (adjacent to the major axes) and testes on the interradial sides of each meridional canal. Specimens 10–100 mm in total length spawn eggs and sperm from paired gonopores between each pair of combs; the spawned eggs are 210–220 µm in diameter (Dunlap-Pianka, 1974). The young ctenophores go through a cydippid stage with tentacle bulbs very near the body surface, as young lobes begin to form. In this stage, the tentacles are long and extensile with many tentilla and are used to feed in the manner of normal cydippid ctenophores. In the young cydippid and earliest lobate forms, all eight adradial canals feed the meridional canals at their aboral ends, but as small lobes are beginning to develop orally, the subtentacular meridional canals begin to elongate below the point where the adradial canals merge with them, and the growing blind-ending extensions of the STMCs become evident.

Geographic distribution. California to British Columbia (the northern limit of this species is unknown).

Vertical Distribution. Surface to 1850 meters depth (Table 1).

**Appendix 2. DNA extraction and Sanger-sequencing protocols.**

The majority of tissues were extracted for Sanger sequencing using the DNeasy Blood and Tissue kit™ (Qiagen, Germantown, MD) following the manufacturer’s protocol. Most extractions included an entire 2 ml tube of tissue. We PCR-amplified and sequenced *COI, H3* and *28S* DNA fragments for *Bolinopsis* lineages worldwide (methods Table S1, localities Table 1, sample sizes Table S2) with the same methods as (Christianson et al., 2022). We also PCR-amplified and sequenced *MDH, LDH,* and *PK* for *B. microptera* and when possible, *B. infundibulum* (Table S2). We designed new primers for the nuclear loci Lactose Dehydrogenase (*LDH*), Malate Dehydrogenase (*MDH*) and Pyruvate Kinase (*PK*) by performing an orthology-guided annotation of the *B. microptera* transcriptome (unpublished data), then running primer3 on the putative *LDH, MDH,* and *PK* coding sequences (JRW,<https://github.com/octopode/orthonaut>). The full length of the locus was then amplified with LongAmp PCR (conditions in Table S10) and sequenced on an ABI3500XL Genetic Analyzer (ThermoFisher Scientific) for eight individuals each from each population.

For some longer genes, internal primers were used to complete the sequencing. Primer sequences, references, and reaction conditions are listed in Table S10. Sequence traces were quality checked and edited within Geneious Prime v2020.2.3.

**Statistical Methods for Sanger Data.**

Sanger-sequenced loci were aligned with Muscle within Geneious Prime. We estimated haplotype networks for each fragment with PopArt v.1.7.2 to illustrate the evolutionary history among *Bolinopsis* species world-wide. We estimated a species tree with *BEAST2 v.2.2.6 for the *COI, 28S,* and *H3* fragments with loci unlinked by partition, a GTR+I+$\Gamma$ site model with empirical frequencies, and an uncorrelated lognormal clock. Mitochondrial fragments were designated as haploid and nuclear fragments as diploid. We used the analytical population size integration model and a Yule tree prior. MCMC analyses were run 200 million generations, with a tree sampled every 10,000 generations and a pre-burnin of 1000 and 10 initialization steps. All *BEAST2 analyses were run multiple times and results viewed in Tracer and Figtree v.1.4.4. Separately but on the same dataset, we estimated a topology with MrBayes v.3.2.7a with a GTR+I+Γ substitution model with three heated and one cold chain that were run for 1,100,000 generations and sampled every 100 generations. The first 100,000 generations were discarded as burn-in. The nuclear loci (*H3, MDH, LDH,* and *PK*) had many heterozygous positions so we resolved them within the program PHASE v.2.1.1 with 10,000 generations of the MCMC chain, burnin of 1000, and six seeds for the random number generator. We calculated summary statistics among lineages, including Kimura two-parameter (K2P) distances, with MegaX v.10.1 and Arlequin v.3.5.2.2. Phased nuclear and mitochondrial data were then reduced to an allelic number code for the programs BA3 v.3.0.4.2 to estimate recent migrants and STRUCTURE v.2.3.4 to estimate population admixture. BA3 analyses were run multiple times for 10^8^ generations, with a sampling interval of 2000 and we discarded the first 10^5^ iterations. We assessed analyses had reached convergence by running analyses multiple times with different random number seeds and reviewing results in TRACER v.1.7. STRUCTURE analyses also were repeated ten times each with the admixture model, uncorrelated allele frequencies and location information as a sample prior. The MCMC chain was run 10^8^ times with a burnin of 10^4^. Posterior probabilities were calculated from a number of populations (*K*) from 2–8. The most probable *K* was then calculated with structure harvester v.web.0.6.94, however K values from 2-6 were plotted.

We estimated demographic histories of populations including the effective population size (*Θμ*), directional migration patterns (*mμ*), population migration rates (*2Neμ*), and time of population split (𝜏𝜇) with IMa3 under a three population model. Mutation rates for genes were unknown so estimates remained scaled by mutation rate (*μ*). Populations were subdivided by locality. Analyses included whole mitochondrial genomes extracted from genomic data and all other Sanger-sequenced, phased gene fragments. IMa is dependent upon several assumptions including no genetic linkage, selective neutrality, gene flow from unsampled populations but is especially vulnerable to recombination within loci. We used IMGC to remove recombining regions of gene fragments and individuals that failed the 4-gamete test to prepare input files. The HKY substitution model was used for all loci. We used the inheritance scalar of 0.25 for mitochondrial and 1.0 for nuclear loci. Analyses were run several times and final analyses included at least 10^8^ steps where the first 10^4^ steps were discarded as a burnin. We applied geometric heating parameters between 0.99 and 0.5 with 50 attempts at chain swapping between the 50-80 chains.

**2 Supplementary Tables**

**Table S1**. Programs and procedures used to estimate and visualize genetic parameters for Sanger data.

| **Program** | **Version** | **Tests and Parameters^3^** | **References** |
| --- | --- | --- | --- |
| Arelequin | 3.5.2.2 | Diversity indices (*H, k, h, π, F_ST_ , Φ_ST_*) | (Escoffier and Lishcer, 2010) |
| BA3 | 3.0.4.2 | Gene flow among populations | (Wilson and Rannala, 2003) |
| *BEAST2 | 2.2.6 | Species tree estimation for *COI, 28S,* and *H3* | (Bouckaert et al., 2019) |
| DNAsp | 6.12.03 | Diversity indices (*S, T_D_, W_Θ_, π, H, H_n_*) | (Rozas et al., 2017) |
| FigTree | 1.4.4 | View phylogenetic consensus trees | http://tree.bio.ed.ac.uk/ |
| Geneious Prime | 2020.2.3 | Sequence editing | https://www.geneious.com |
| Genodive | 3.05 | *G_IS_, F_ST_*, parentage, hybrid index, and *HWE* | (Miermans, 2020) |
| HybridCheck | 1.0 | ABBA-BABA for Sanger loci | (Ward and van Oosterhout, 2016) |
| IMa3 | 3.0 | Demographic parameters (*Θμ,* *mμ,* *2Nemμ,* 𝜏𝜇) | (Hey, 2010) |
| IMGC |  | Recombination | (Woerner et al., 2007) |
| MegaX | 10.1 | K2P distances and summary statistics | (Kumar et al., 2018) |
| Muscle |  | Sequence alignment | (Edgar, 2004) |
| MrBayes | 3.2.7a | Bayesian phylogeny estimation | (Ronquist et al., 2012) |
| PopArt | 1.7.2 | Minimum spanning network estimation | (Bandelt et al., 1999) |
| PHASE | 2.2.1 | Resolution of heterozygous loci | (Stephens and Donnelly, 2003) |
| Primer3 |  | Primer design | (Untergasser et al., 2012) |
| R | 4.1.0 | Framework for statistical estimation | (Team, 2017) |
| RStudio | 1.4.1717 | Work environment for R | (Team, 2015) |
| STRUCTURE | 2.3.4 | Assignment test (*K*) | (Pritchard et al., 2000) |
| STRUCTURE HARVESTER | web.0.6.94 | Estimating (*K*) probability | (Earl and vonHoldt, 2012) |
| Tidyverse |  | Visualizing boxplots, SFS and admixture results and estimating tSNE | (Wickham et al., 2019) |
| TRACER | 1.7 | Phylogenetic convergence | (Rambaut et al., 2018) |

^1^ Parameters: *H,* number of haplotypes; *k*, number of polymorphic sites; *h,* haplotype diversity; π, nucleotide diversity per site; *F_ST_*, standardized molecular variance among populations; *ϕ_ST_*, standardized molecular variance among populations; *S=*segregating sites; *D,* Tajima’s D; *W_Θ_,* Waterson’s *Θ;* π*,* nucleotide diversity; *H,* haplotype diversity; *H_n,_* Fay and Wu’s H; *E,* Ewens-Watterson estimator; overall DHEW compound test; *G_IS,_* inbreeding; *HWE,* Hardy-Weinberg Equilibrium; *tSNE,* t-distributed stochastic neighbor embedding; *SFS*, site-frequency spectrum; *Θμ,* effective population size; *mμ,* immigration; *2Nemμ,* population migration; 𝜏𝜇, time to the most recent common ancestor (all scaled by 𝜇; mutation rate); *K,* number of genotypic clusters.

**Table S2.** Sample sizes of each species from each population sequenced for Sanger genetic analyses. – indicates no sequence, nuclear loci were phased and represent biallelic data in analyses.

| **Species** | **Population** | **Locus** | | | | | |  |
| --- | --- | --- | --- | --- | --- | --- | --- | --- |
|  |  | *COI* | *H3* | *LDH1* | *LDH2* | *MDH* | *PK* | *28S* |
| *B. ashleyi* | Australia | 1 | 1 | - | - | - | - | 1 |
| *B. infundibulum* | MA | 13 | 3 | 2 | 2 | - | 2 | - |
| *B. microptera* | WA | 30 | 6 | 16 | 14 | 4 | 21 | - |
| *B. microptera* | NorCal | 142 | 42 | 42 | 58 | 6 | 54 | - |
| *B. microptera* | SoCal | 18 | 2 | - | 10 | 17 | 16 | - |
| *B. microptera* | WAxCA | 192 | 15 | 14 | 14 | 15 | 22 | - |
| *B. mikado* | Japan | 5 | 5 | - | - | - | - | 5 |
| *Bolinopsis* n.sp. | Australia | 1 | 1 | - | - | - | - | 1 |
| *B.* aff. *vitrea* | Hawaii | 17 | 2 | - | - | - | - | 2 |
| *B. vitrea* | Bahamas | 3 | 3 | - | - | - | - | 3 |
| *M. leiydi* | FL | 2 | 2 | - | - | - | - | 2 |

**Table S3.** Pairwise *K2P* distances calculated within and between named and un-named species and populations of *Bolinopsis* and *Mnemiopsis leidyi* for the *COI* fragment and for whole mitochondrial genomes (bold values).

|  | *infun* | WA | NorCal | SoCal | *ashleyi* | AUnsp | *mikado* | *vitrea* | *Mnem* | Moorea | HI |
| --- | --- | --- | --- | --- | --- | --- | --- | --- | --- | --- | --- |
| *infun* | 0.031 |  |  |  |  |  |  |  |  |  |  |
| WA | 0.118 | 0.001  **0.001** |  |  |  |  |  |  |  |  |  |
| NorCal | 0.109 | 0.036  **0.023** | 0.029  **0.021** |  |  |  |  |  |  |  |  |
| SoCal | 0.110 | 0.036  **0.025** | 0.036  **0.021** | 0.032  **0.022** |  |  |  |  |  |  |  |
| *ashleyi* | 0.173 | 0.182 | 0.182 | 0.177 | nc |  |  |  |  |  |  |
| AU n. sp. | 0.206 | 0.206 | 0.206 | 0.202 | 0.142 | nc |  |  |  |  |  |
| *mikado* | 0.201 | 0.200 | 0.199 | 0.196 | 0.142 | 0.041 | 0.003 |  |  |  |  |
| *vitrea* | 0.206 | 0.202 | 0.201 | 0.197 | 0.134 | 0.113 | 0.123 | 0.003 |  |  |  |
| *Mnem* | 0.210 | 0.214 | 0.213 | 0.209 | 0.115 | 0.131 | 0.136 | 0.134 | nc |  |  |
| Moorea | 0.221 | 0.210 | 0.210 | 0.209 | 0.150 | 0.132 | 0.145 | 0.109 | 0.146 | nc |  |
| Hawaii | 0.243 | 0.219 | 0.219 | 0.209 | 0.159 | 0.140 | 0.156 | 0.114 | 0.160 | 0.027 | 0.015 |

**Table S4**. Fixed differences (highlighted in gray) for the 648 bp alignment for *M. leidyi* and *Bolinopsis* species including *B. infundibulum* (*infun*), *B. microptera* – WA lineage (*mic*WA), *B. microptera* – CA lineage (*mic*CA), *B. ashleyi* – AU (*ashleyi*), *Bolinopsis* n. sp – AU (n.sp.AU), *B. mikado –* Japan (*mikado*), *M. leidyi* – Florida (*Mnem*), *B. vitrea* – Bahamas (*vitrea*), *B.* aff. *vitrea* – Moorea, and *B.* aff. *vitrea* – Hawaii.

| species | 59 | 101 | 134 | 156 | 163 | 166 | 170 | 189 | 200 | 270 | 327 | 423 | 479 | 489 | 566 | 587 | 593 | 599 |
| --- | --- | --- | --- | --- | --- | --- | --- | --- | --- | --- | --- | --- | --- | --- | --- | --- | --- | --- |
| *infun* | C | T/G | T | A | T | T | T | G/A | T/C | A | T | T | T | T | A | A | C | A |
| *mic*WA | T | T | T | A/G | T | T | T | A | T | T | T | T | T | T | A | T | T | T |
| *mic*CA | T | T | T | A | T/C | T | T/C | A | T | T | T | T | T/G | T | A | T | T | T |
| *ashleyi* | T | T | T | T | G | A | T | A | T | T | A | T | T | T | A | A | T | T |
| n.spAU | T | T | T | A | T | T | T | A | A | T | T | T | T | T | G | A | T | T |
| *mikado* | T | T | T | A | T | T | T | A | G | T | T | T | T | T | A | A | T | T |
| Mnem | T | T | A | A | A | G | A | A | C | T | A | T | T | T | A | A | T | T |
| *vitrea* | T | C | T | A | T | T | T | A | T | T | T | T | T | T | A | C | T | T |
| Moorea | T | T | T | A | T | T | T | C | T | T | G | A | A | C | T | T | T | T |
| Hawaii | T | T | T | A | T | T | T/C | C | T | T | G | A | A | C | T | T | T | T |

**Table S5**. Diversity statistics and compound neutrality tests for *B. microptera* from eastern Pacific populations for mitochondrial genomes including *S=*segregating sites*, T_D_ =* Tajima’s D, *WΘ =* Waterson’s *Θ*, π *=* nucleotide diversity, *H* = haplotype diversity, *H_n_ =* Fay and Wu’s H, *E =* Ewens-Watterson estimator, and the overall DHEW compound test with corresponding ± confidence intervals or P-values in parentheses. Significant results in bold.

| **Population** | ***S*** | ***T_D_*** | ***W_θ_*** | ***π*** | ***H*** | ***H_n_*** | ***E*** | ***P_DHEW_*** |  |
| --- | --- | --- | --- | --- | --- | --- | --- | --- | --- |
| WA | 19 | **-1.8** | 0.001 | 0.0006 | 0.9 | **-2.2** | 0.8 | **0.009** |  |
|  |  | (0.01) | (0.0002) | (0.0003) | (0.08) | (0.02) | (0.8) |  |  |
| NorCal | 517 | 0.36 | 0.018 | 0.02 | 1.0 | **-2.4** | 2.8 | 0.5 |  |
|  |  | (0.7) | (0.0007) | (0.0009) | (0.02) | (0.02) | (1.0) |  |  |
| SoCal | 479 | 0.23  (0.7) | 0.019  (0.0007) | 0.02  (0.002) | 1.0  (0.02) | **-2.0**  (0.03) | 2.4  (1.0) | 0.4 |  |

**Table S6**. Top diagonal includes weighted pairwise F_ST_ values estimated nuclear genomic data. Bottom diagonal includes pairwise F_ST_ values calculated in GenoDive for *COI, H3, LDH1, LDH2, MDH,* and *PK* allele frequencies. Significant differences indicated by bolded values (P$\leq0.001)$. Populations in order of decreasing latitude including; WA: Friday Harbor, WA, NorCal: Monterey Bay area CA, SoCal: San Luis Obispo–Channel Islands, CA, and WAxCA: progeny of *Bolinopsis* from Washington crossed with NorCal.

| F_ST_ | WA | NorCal | SoCal |
| --- | --- | --- | --- |
| WA | -- | 0.234 | 0.287 |
| NorCal | **0.130** | -- | 0.034 |
| SoCal | **0.218** | 0.006 | -- |
| WAxCA | **0.285** | **0.104** | **0.160** |

**Table S7**. Maximum likelihood estimates (MLE) of demographic population parameters and low (L) and highest (H) 95% posterior density confidence intervals for mean effective population sizes (*θ*), time of population splitting (*t*), and immigration rates (*m*) all scaled by mutation rate (*μ*) for populations of *B. microptera* from; WA: Friday Harbor, WA, NorCal: Monterey Bay area CA, SoCal: San Luis Obispo–Channel Islands, CA. Estimates were made with Sanger-sequenced data. ← denotes the direction of migration (from a population into another). Bolded values indicate significant LLR test.

| Parameter | *MLE* | L | H |
| --- | --- | --- | --- |
| θ_WA_ | 17.4 | 0.2 | 213 |
| θ_NorCal_ | 202.9 | 3.8 | 399 |
| θ_SoCal_ | 215.7 | 3 | 399 |
| θ_A_ | 233 | 58.2 | 400 |
| *t* | 4.8 | 0 | 12.6 |
| *m*_WA ←NorCal_ | 0.3 | 0 | 1.45 |
| *m*_NorCal←WA_ | 0.08 | 0 | 0.85 |
| *m*_WA←SoCal_ | **0.7** | **0** | **3.7** |
| *m*_SoCal←WA_ | 0.1 | 0 | 0.95 |
| *m*_NorCal←SoCal_ | **3.73** | **0** | **13.5** |
| *m*_SoCal←NorCal_ | **3.13** | **0** | **11.65** |
| *m*_←ghost_ | **8.45** | **0.4** | **31.1** |

**Table S8**. Results of BA3 analyses with the neutral polymorphic markers (*COI, H3, LDH1, LDH2, MDH,* and *PK*) with SD in parentheses for migration rates and SE for inbreeding (F_STAT_) estimates for *B. microptera* populations.

|  | SOURCE | | | | | F_STAT_ |
| --- | --- | --- | --- | --- | --- | --- |
| DESTINATION |  | WA | NorCal | SoCal | WAxCA |  |
|  | WA | 0.93 (0.04) | 0.048  (0.035) | 0.012  (0.012) | 0.012  (0.012) | 0.40 (0.05) |
|  | NorCal | 0.004  (0.004) | 0.988  (0.007) | 0.004  (0.004) | 0.004  (0.004) | 0.32 (0.02) |
|  | SoCal | 0.032  (0.003) | 0.239  (0.04) | 0.706  (0.03) | 0.024  (0.02) | 0.44 (0.21) |
|  | WAxCA | 0.017  (0.017) | 0.284  (0.03) | 0.016  (0.015) | 0.683  (0.02) | 0.46 (0.25) |

**Table S9**. Results from ABBA-BABA tests (*D* statistics) for *LDH2* and *PK*. *D* = Patterson’s D, and *p-*values calculated by Fisher’s combined probability test. *D* > 0 gene flow between P2 and P3, *D* < 0 gene flow between P1 and P3. Significant gene flow between populations indicated in bold. All *Bolinopsis* sp. collected in California were combined.

| **Locus** | **Outgroup** | **P3** | **P2** | **P1** | **#ABBA** | **#BABA** | ***D*** | ***p-value*** |
| --- | --- | --- | --- | --- | --- | --- | --- | --- |
| *LDH2* | *infun* | WA | CA | WAxCA | 1 | 2 | -0.125 | 0.5 |
|  | *infun* | CA | WAxCA | WA | 6 | 1 | 0.915 | 0.06 |
| *PK* | *infun* | **WAxCA** | **CA** | WA | 29 | 3 | 0.884 | 0.00001 |
|  | *infun* | **CA** | **WAxCA** | WA | 29 | 10 | 0.574 | 0.001 |

**Table S10**. PCR primer sequences and reaction conditions.

| Locus | Primer Sequence 5’→ 3’ | Methods | Length | References |
| --- | --- | --- | --- | --- |
| *28S* rDNA | F63: AGCGGAGGAAAAGAAACTA  R1091: TACTAGAAGGTTCGATTAGTC | COI50 | ~1100 bp | (Sonnenberg et al., 2007) |
| Cytochrome-C-oxidase subunit I | F259MBBoli: GCCGATATGTGCTTGCCACG  R1060: ACCRGAYARGCCACCAAAAGT | COI50 | ~800 bp | (Christianson et al., 2022) |
| *COI* | F259Mod5: GCNGATATGTGYYTNCCNMG | COI50 | ~800 bp | (Christianson et al., 2022) |
| Histone-3  (*H3*) | H3F: ATGGCTCGTACCAAGCAGACVGC  H3R: ATATCCTTRGGCATRATRGTGAC | Phusion Fast 58 | ~380 bp | (Colgan et al., 2000) |
| Lactose Dehydrogenase  (*LDH*) | BinfLDH\|v6-26265F: GAGAACCTGTATTTCCAGATGTTCAGATTACCGGCAGCT  BinfLDH\|v6-26265R: CACTGAGCCTCCACCTAGCCTCTGGATGTGGGCACAGAGTT | LongAmp | TX: 1650 bp, AF: ~12 kb | Designed by JRW |
| *LDH*-F1 | BinfLDH\|v6-26265F: GAGAACCTGTATTTCCAGATGTTCAGATTACCGGCAGCT  BLDH329R: CACGGGTAGGGTGGAATGAC | Phusion Fast 58 | ~330 bp | This study |
| *LDH*-F2 | BLDH329F: GTCATTCCACCCTACCCGTG  BLDH1112R: AGGGAAATGAAGGCCGTCAG | Phusion Fast 58 | ~760 bp | This study |
| Malate Dehydrogenase  (*MDHc*) | BinfMDHc\|v6-06164.p0F: GAGAACCTGTATTTCCAGATGGCTACGAACAAGAGTCCT  BinfMDHc\|v6-06164.p0R: CACTGAGCCTCCACCTAGCCTTTAGTTGTCGGCTTCAGCGG | LongAmp | TX: 1020 bp, AF: ~12 kb | Designed by JRW |
| MDHc-F1 | BinfMDHC\|v6-06164.p0F/  BoliMDH-527R: TCGTCAGTAGCCACAACACC | Phusion Fast 58 | ~530 bp | This study |
| MDHc-F2 | BoliMDH424F: CAC ATG GCT TTG CAG TGC TT  BoliMDH873R: CAG TCA CAT GTC AAC GTC ACC | Phusion Fast 58 | ~450 bp | This study |
| Pyruvate Kinase (*PK*) | BinfPK\|v6-10519.p0F: GAGAACCTGTATTTCCAGATGGGGTCGCTGTTAAAAGA  BinfPK\|v6-10519.p0R: CACTGAGCCTCCACCTAGCCTGCATAAACGCGGTTCGAACA | Phusion stepUP, 65 | TX: 1697 bp, AF: ~12 kb | Designed by JRW |
| *PK-F1* | BoliPK: GAGAACCTGTATTTCCAGATGGGGTCGCTGTTAAAAGA  BPK219R: GCTCCTTTAGCGGTCTCTCC | Phusion Fast 58 | 219 bp | This study |
| *PK-F2* | BPK162F: ATGTTGTCAGGAGAGACCGC  BPK623R: CCACGGCATCTCTTCCTCTC | Phusion Fast 58 | ~1000 bp | This study |

*COI50*: Amplitaq Gold 360 DNA polymerase, 95°C 10’ (94° C 1’, 50°C 1’, 72°C 1’) x 35, 72°C 5’, 4°C hold ThermoFisher; *LongAmp*: LongAmp® Taq, NEB; 94°C 30s (94° C 30s, 65°C 1’, 65°C 10’) x 35, 65°C 10’, 4°C hold 2. *PhusionFast58*: Phusion®High-Fidelity DNA polymerase, NEB; 98°C 5’ (98° C 15s, 58°C 30s, 72°C 30s) x 35, 72°C 5’, 4°C hold. 3. *PhusionstepUP* 65: Phusion®High-Fidelity DNA polymerase, NEB, 98°C 5’ (98° C 15s, 58°C 30s, 72°C 30s) x 35, 72°C 5’, 4°C hold

**References**

Bandelt, H., Forster, P., and Röhl, A. (1999). Median-Joining networks for inferring intraspecific phylogenies. *Molecular Biology and Evolution* 16**,** 37-48.

Bouckaert, R., Vaughan, T.G., Barrido-Sottani, J., Duchêne, S., Fourment, M., Gavryushkina, A., and Al., E. (2019). BEAST 2.5: An advanced software platform for Bayesian evolutionary analysis. *PLoS comuptational biology* 15**,** e1006650.

Christianson, L.C., Johnson, S.B., Schultz, D.T., and Haddock, S.H.D. (2022). Hidden diversity of Ctenophora revealed by new mitochondrial *COI* primers and sequences. *Molecular Ecology Resources* 22**,** 283-294.

Colgan, D.J., Ponder, W.F., and Eggler, P.E. (2000). Gastropod evolutionary rates and phylogenetic relationships assessed using partial 28S rDNA and histone H3 sequences *Zoologica Scripta* 29**,** 29-63.

Dunlap-Pianka, H. (1974). "Ctenophora," in *Reproduction of Marine Invertebrates,* eds. A. Giese & J. Pearse. (New York: Academic Press), 201-265.

Earl, D.A., and Vonholdt, B.M. (2012). STRUCTURE HARVESTER: a website and program for visualizing STRUCTURE output and implementing the Evanno method. *Conservation Genetics Resources* 4**,** 359-361.

Edgar, R. (2004). MUSCLE: multiple sequence alignment with high accuracy and high throughput. *Nucleic Acids Research* 32**,** 1792-1797.

Escoffier, L., and Lishcer, H.E.L. (2010). Arelequin suite ver 3.5: A new series of programs to perform population genetics analyses under Linux and Windows. *Molecular Ecology Resources* 10**,** 564-567.

Hey, J. (2010). Isolation with migration models for more than two populations. *Molecular Biology and Evolution* 27**,** 905-920.

Kumar, S., Stecher, G., Li, M., Knyaz, C., and Tamura, K. (2018). MEGA X: Molecular Evolutionary Genetics Analysis across computing platforms. *Molecular Biology and Evolution* 35**,** 1547-1549.

Miermans, P.G. (2020). GENODIVE version 3.0: Easy-to-use software for the analysis of genetic data of diploids and polyploids. *Molecular Ecology Resources* 20**,** 1126-1131.

Pritchard, J.K., Stephens, M., and Donnelly, P. (2000). Inference of population structure using multilocus genotype data. *Genetics* 155**,** 945-959.

Rambaut, A., Drummond, A.J., Xie, D., Baele, G., and Suchard, M.A. (2018). Posterior summarization in bayesian phylogenetics using Tracer 1.7. *Systematic Biology* 67**,** 901-904.

Ronquist, F., Teslenko, M., Van Der Mark, P., Ayres, D.L., Darling, A., Höhna, S., Larget, B., Liu, L., Suchard, M.A., and Huelsenbeck, J.P. (2012). MrBayes 3.2: Efficient bayesian phylogenetic inference and model choice across a large model space. *Systematic Biology* 61**,** 539-542.

Rozas, J., Ferrer-Mata, A., Sánchez, D., Jc, Guirao-Rico, S., Librado, P., Ramos-Onsins, S., and Sánchez-Gracia, A. (2017). DnaSP 6: DNA sequence polymorphism analysis of large datasets. *Molecular Biology and Evolution* 34**,** 3299-3302.

Sonnenberg, R., Nolte, A.W., and Tautz, D. (2007). An evaluation of LSU rDNA D1-D2 sequences for their use in species identification. *Frontiers in Zoology* 4.

Stephens, M., and Donnelly, P. (2003). A comparison of Bayesian methods for haplotype reconstruction from population genotype data. *American Journal of Human Genetics* 73**,** 1162-1169.

Team, R. (2015). "RStudio: Integrated Development for R". (Boston, MA: RStudio Inc).

Team, R.C. (2017). "R: A language and environment for statistical computing". (Vienna, Austria: R Foundation for Statistical Computing).

Untergasser, A., Cutcutache, I., Koressaar, T., Ye, J., Faircloth, B.C., Remm, M., and Rozen, S.G. (2012). Primer3--new capabilities and interfaces. *Nucleic acids research* 40**,** e115.

Ward, B.J., and Van Oosterhout, C. (2016). HybridCheck: software for rapid detection, visualization and dating of recombinant regions in genome sequence data. *Molecular Ecology Resources* 16**,** 534-539.

Wickham, H., Averick, M., Bryan, J., Chang, W., Mcgowan, L.D.A., François, R., Grolemund, G., Hayes, A., Henry, L., Hester, J., Kuhn, M., Pedersen, T.L., Miller, E., Bache, S.M., Müller, K., Ooms, J., Robinson, D., Seidel, D.P., Spinu, V., Takahashi, K., Vaughan, D., Wilke, C., Woo, K., and Yutani, H. (2019). Welcome to the tidyverse. *Journal of Open Source Software* 4**,** 1686.

Wilson, G.A., and Rannala, B. (2003). Bayesian inference of recent migration rates using multilocus genotypes. *Genetics* 163**,** 1177-1191.

Woerner, A.E., Cox, M.P., and Hammer, M.F. (2007). Recombination-filtered genomic datasets by information maximization. *Bioinformatics* 23**,** 1851-1853.
