# Supplementary material for: Speciation of pelagic zooplankton: Invisible boundaries can drive isolation of oceanic ctenophores
Source: Front Genet. 2022 Oct 7;13:970314. doi: 10.3389/fgene.2022.970314 (PMC9585324; doi:10.3389/fgene.2022.970314)
Supplement: Supplementary file 3 [file Image4.pdf]

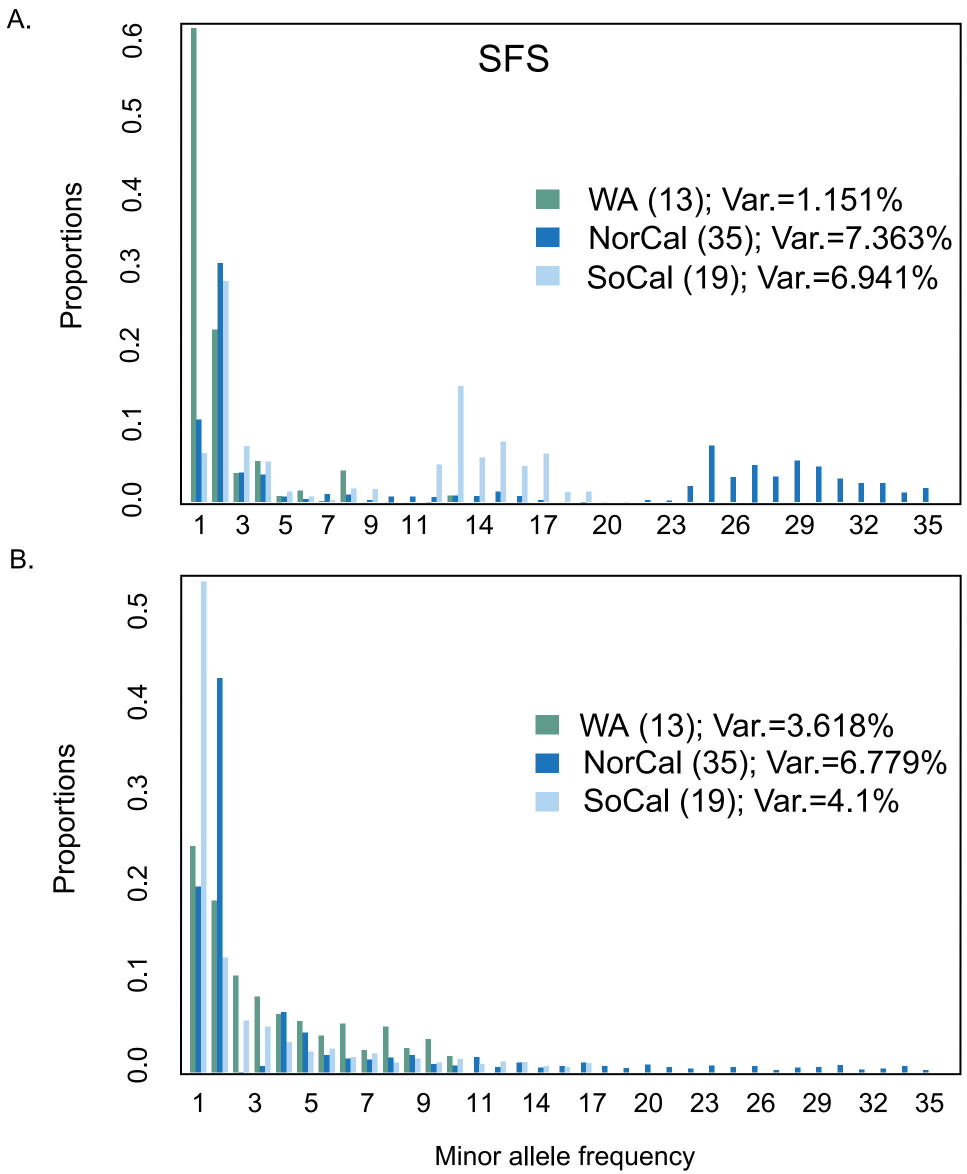

Figure S4. Folded proportional site frequency spectra (SFS) for minor allele frequencies (MAF) for WA (green), NorCal (blue), and SoCal (light blue) populations for (A) Mitochondrial genomic data and (B) Nuclear genomic data of *B. microptera.*
