# Supplementary material for: Speciation of pelagic zooplankton: Invisible boundaries can drive isolation of oceanic ctenophores
Source: Front Genet. 2022 Oct 7;13:970314. doi: 10.3389/fgene.2022.970314 (PMC9585324; doi:10.3389/fgene.2022.970314)
Supplement: Supplementary file 5 [file Image3.pdf]

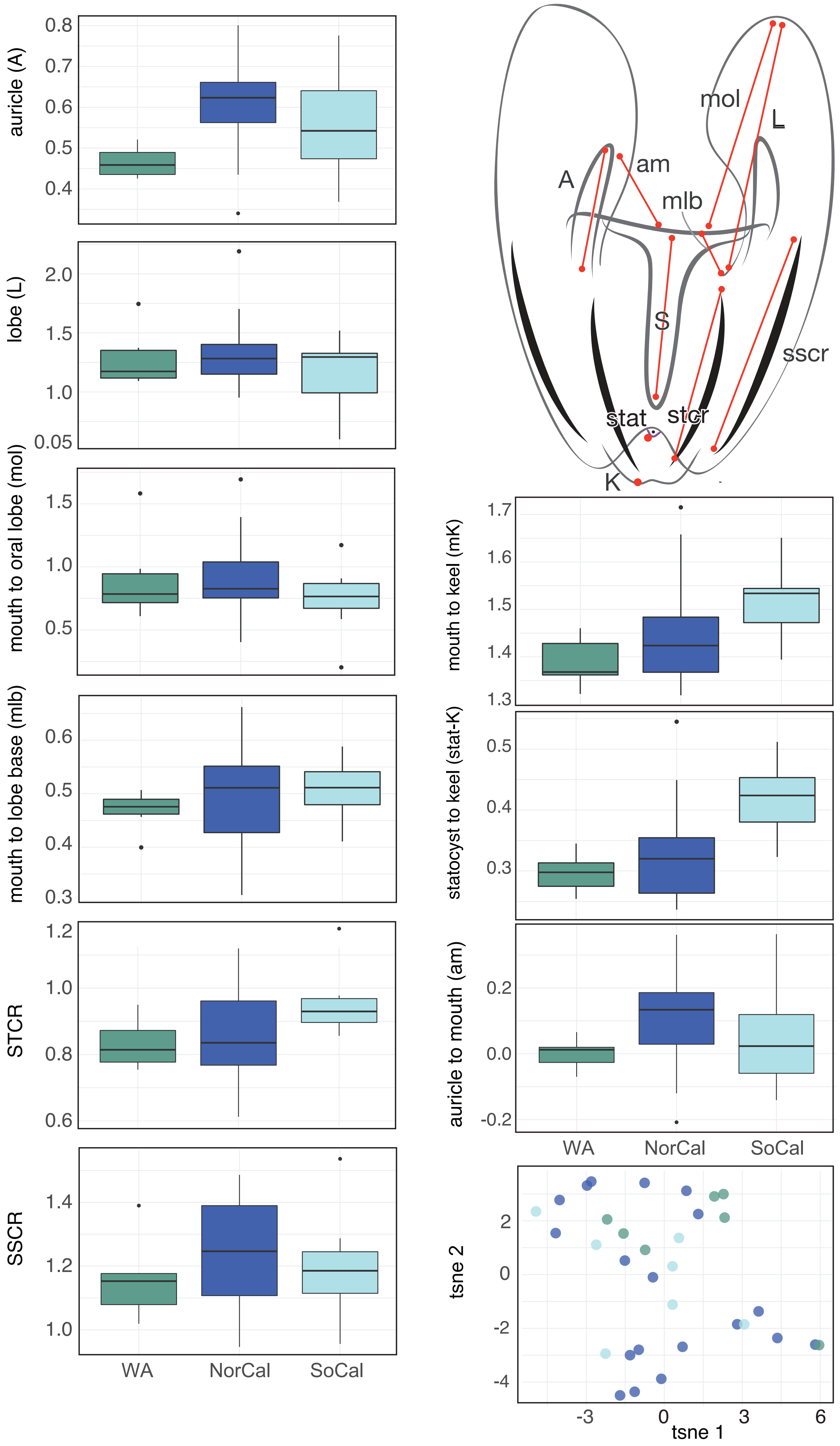

Figure S3. Boxplots of morphological measurements among *Bolinopsis microptera* populations including Washington (WA, green), Monterey Bay area (NorCal, blue), and from San Luis Obispo to the Channel Islands, CA (SoCal, light blue) for auricle (A) and lobe (L) lengths, the distances from the mouth to the oral lobe (mol), mouth to the lobe base (mlb), mouth to keel (mK), statocyst to keel (stat-K), the auricle tip to the mouth (am), and the lengths of the sub-tentacular ctene row (stcr) and sub-stomodaeal ctene row (sscr) and tSNE results of combined measurements for each population.
