# Supplementary material for: Speciation of pelagic zooplankton: Invisible boundaries can drive isolation of oceanic ctenophores
Source: Front Genet. 2022 Oct 7;13:970314. doi: 10.3389/fgene.2022.970314 (PMC9585324; doi:10.3389/fgene.2022.970314)
Supplement: Supplementary file 7 [file Image1.pdf]

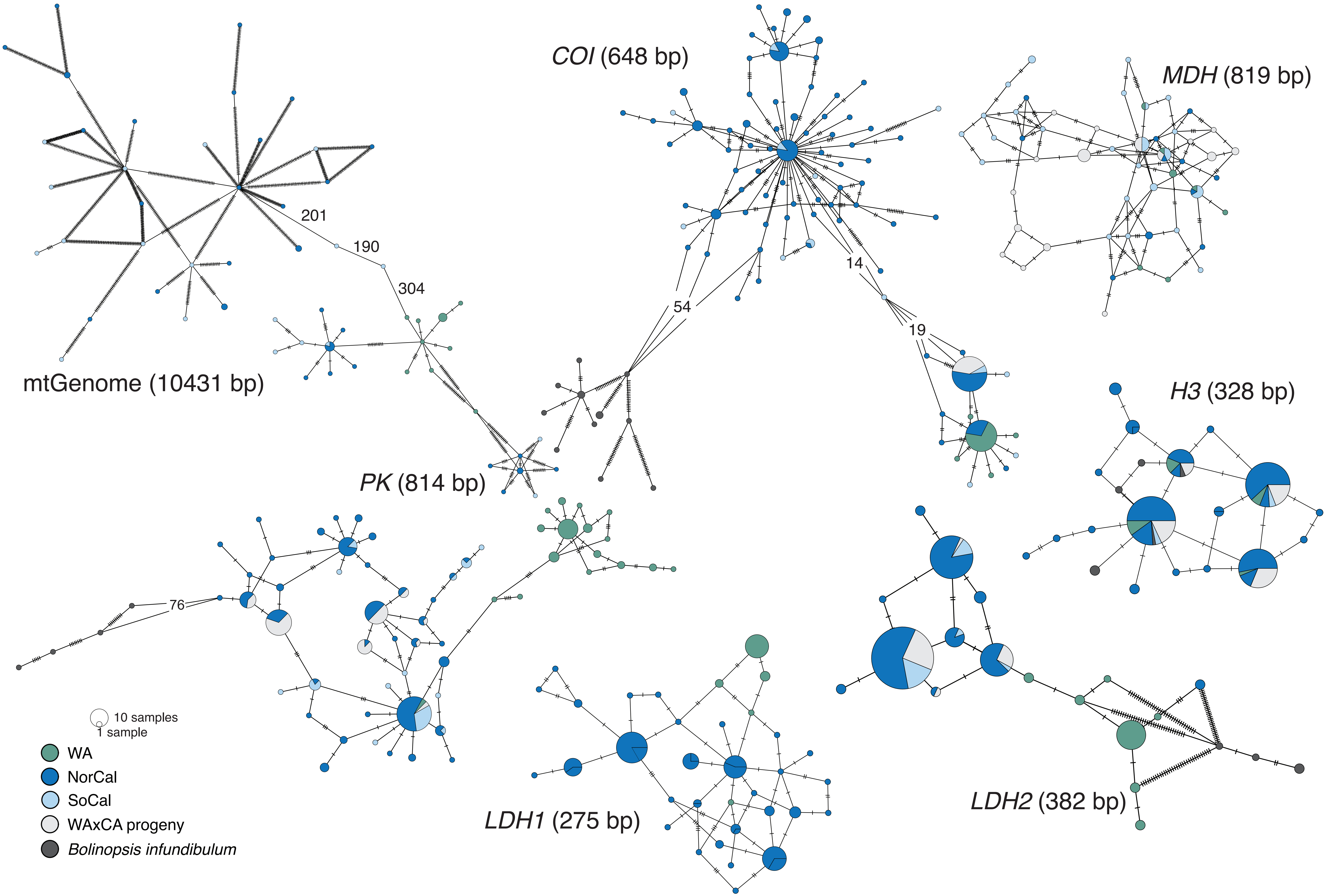

Figure S1. Minimum spanning haplotype networks for the mitochondrial genomes (10431 bp), a fragment of mitochondrial *COI* (648 bp), and nuclear fragments of *MDH* (819 bp), *PK* (814 bp), *H3* (328 bp), *LDH1* (275 bp) and *LDH2* (382 bp) for Bolinopsis sequenced from Friday Harbor, WA (WA, green), the Monterey Bay area, CA (NorCal, blue), and San Luis Obispo–Santa Barbara Basin, CA (SoCal, light blue), progeny of the WAxCA experiment (gray), and *B. infundibulum* s.s. (dark gray). Dashes or numbers on branches represent mutations.
